# Chiral Phonons and Anomalous Excitation-Energy-Dependent Raman Intensities in Layered AgCrP_2_Se_6_

**DOI:** 10.1021/acsnano.5c00381

**Published:** 2025-07-14

**Authors:** Rahul Rao, Jie Jiang, Ruth Pachter, Thuc T. Mai, Valentine Mohaugen, Maria F. Muñoz, Ryan Siebenaller, Emmanuel Rowe, Ryan Selhorst, Andrea N. Giordano, Angela R. Hight Walker, Michael A. Susner

**Affiliations:** † Materials and Manufacturing Directorate, Air Force Research Laboratory, Wright-Patterson Air Force Base, Ohio 45433, United States; ‡ Blue Halo Inc., Dayton Ohio 45434, United States; § Quantum Measurement Division, Physical Measurement Laboratory, National Institute of Science and Technology, Gaithersburg, Maryland 20899, United States; ∥ Department of Materials Science and Engineering, The Ohio State University, Columbus, Ohio 43210, United States; ⊥ National Research Council, Washington, D.C. 20001, United States

**Keywords:** 2D material, chiral phonons, chirality, circularly polarized Raman, helical modes, van der Waals material

## Abstract

Structural anisotropy in layered two-dimensional materials can lead to highly anisotropic optical absorption which, in turn, can profoundly affect their phonon modes. These effects include lattice orientation-dependent and excitation energy-dependent mode intensities that can enable next-generation phononic and optoelectronic applications. Here, we report anomalous Raman spectra in single-crystalline AgCrP_2_Se_6_, a layered antiferromagnetic material. Density functional theory calculations and experimental measurements reveal several features in the Raman spectra of bulk and exfoliated AgCrP_2_Se_6_ crystals including three chiral phonon modes. These modes exhibit large Raman optical activities (circular intensity differences) in bulk AgCrP_2_Se_6_, which progressively decrease with thickness. We also observe strong excitation-energy-dependent peak intensities as well as a decrease in anti-Stokes peak intensities at room temperature with increasing excitation energy, resulting in an apparent cooling by up to 220 K. All of these anomalies in bulk and exfoliated flakes are attributed to 1) the ABC layer stacking structure of AgCrP_2_Se_6_ and 2) the more constrained metal ion environment in the Se-bounded octahedral cage, causing hybridization between the Se and Ag/Cr electron densities and resulting in charge transfer that strongly affects the electron–phonon coupling. Consequently, this work positions AgCrP_2_Se_6_ as an exciting two-dimensional material for optical and phononic applications.

## Graphical abstract

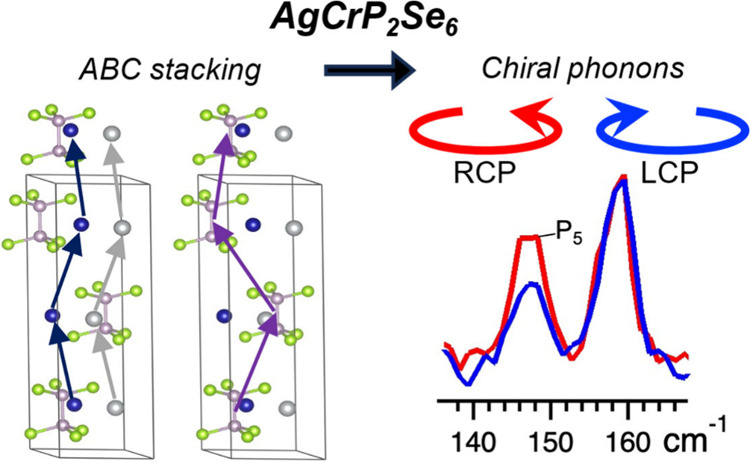

## Introduction

Structural anisotropy in layered two-dimensional (2D) materials can lead to highly anisotropic optical absorption, enabling new ways to tune light-matter coupling through the excitation, detection, and control of light along various crystallographic axes.^1–6^ Some of the consequential effects are hyperbolic plasmons and excitons,^7,8^ giant nonlinear second harmonic generation,^9^ as well as mechanical anisotropy.^10^ These properties can be leveraged in various photonic, phononic, and optoelectronic devices wherein the 2D materials are incorporated as the active layer. Examples of applications using such devices include optical birefringent polarizers, polarized light emitting diodes and lasers, polarization sensitive photodetectors, and thermoelectrics.^2,11^

The anisotropic optical response of 2D materials can be readily measured with angle-resolved linearly polarized Raman scattering. Prior studies have revealed significant lattice-orientation-dependent mode intensities in materials like black phosphorus,^12,13^ ReSe_2_,^14,15^ MoO_3_,^16^ As_2_S_3_,^17^ and WTe_2_.^18^ The Raman peak intensities can also depend on the energy/wavelength of the excitation laser and can be studied using resonance Raman scattering where the incident photon absorption results in a real electronic transition rather than a virtual transition as in the normal Raman scattering process. Mode-specific excitation-energy-dependent intensities were found for Raman-active Stokes and anti-Stokes modes in ReS_2_ ^19^ and MoTe_2_ ^20,21^ and have been attributed to larger contributions of electron–phonon coupling for specific phonon modes compared to electron–photon interaction. In addition, circularly polarized Raman scattering studies have revealed large differences in the intensities of certain phonon modes in ReS_2_ and ReSe_2_ with either left or right circularly polarized (LCP and RCP, respectively) excitations.^22^ These differences were attributed to quantum interference between the first-order Raman scattering processes at different *k* points in the Brillouin zone.^23^ Ultrafast spectroscopy measurements have also revealed chiral phonons (helical or circular vibrating phonon modes associated with angular momenta) at the high-symmetry zone edges in monolayer 2D materials.^24^ In short, optical anisotropies may affect the lattice and vibrational modes of 2D materials in different ways, allowing for tunability of the electron–phonon coupling in individual materials and their heterostructures.

Here, we report anomalous Raman spectra in single-crystalline AgCrP_2_Se_6_, which is a layered antiferromagnetic material (*T*_N_ ∼ 42 K)^25^ belonging to the 2D metal phosphorus chalcogenide/metal thio- and selenophosphate family.^26^ Using both theoretical calculations and experimental measurements, we have revealed several unique features in the Raman spectra of bulk and exfoliated AgCrP_2_Se_6_ crystals including three chiral phonon modes. These modes exhibit large Raman optical activities (circular intensity differences) in bulk AgCrP_2_Se_6_, which progressively decrease with thickness. We also observed variations in peak intensities in the bulk crystal and exfoliated flakes, some of which are maximum at our lowest excitation energy (1.58 eV) and others maximized at higher excitation energies (2.33 or 2.41 eV). Finally, we observed a decrease in anti-Stokes peak intensities at room temperature with increasing excitation energy, resulting in an apparent cooling by up to 220 K. We attribute our observations to the structural anisotropy in AgCrP_2_Se_6_. The chiral modes arise from the ABC layer stacking and the anomalous peak intensities are tentatively attributed to the smaller octahedral volume for the metal cations that in turn causes hybridization between the Se and Ag/Cr electron densities, and, consequently, charge transfer and variations in electron–phonon coupling.

## Results and Discussion

### Observation of Chiral Phonons

Each layer of AgCrP_2_Se_6_ consists of a [P_2_Se_6_]^4–^ framework in which the Se atoms are positioned on the vertices of Se_6_ octahedra. One third of these octahedral sites contain P–P dimers, which support the anionic sublattice; the remaining two thirds of the octahedral sites are occupied by hexagonally arranged and alternating Ag^+^ and Cr^3+^ cations (which charge-balance the structure). Our previous study^25^ showed the crystal structure of AgCrP_2_Se_6_ to be trigonal, belonging to the *P*3_1_12 (No. 151) space group. The schematic in Figure 1a shows four layers (one unit cell plus an additional layer), each with a [P_2_Se_6_]^4–^ unit and Ag^+^ and Cr^3+^ ions. Note that *P*3_1_12 is a chiral space group and that inorganic materials in this space group can exhibit structural chirality^27^ as well as chiral phonons.^28^ Indeed, a helical arrangement of the Ag^+^ and Cr^3+^ ions can be seen in the structure along the layer stacking direction (denoted by the arrows in Figure 1a). Further confirmation of the helical atomic arrangement can be seen in a top-down view of the Ag^+^ and Cr^3+^ ions as well as a [P_2_Se_6_]^4–^ unit, which shows *C*_3_ rotational symmetry (Figure S1). The alternating hexagonal arrangement of metal cations is similar to other Ag- and Cu-based metal selenophosphates. However, a key difference between AgCrP_2_Se_6_ and related materials is the stacking sequence between the layers, which is ABC as can be seen in Figure 1a. The ABC stacking sequence can also be found in quaternary CuAlP_2_Se_6_ ^29^ and ternary In_4/3_P_2_S_6_,^30^ Cd_2_P_2_Se_6_, Fe_2_P_2_Se_6_, Mg_2_P_2_Se_6_, Mn_2_P_2_Se_6_, and Zn_2_P_2_Se_6_.^31^ Related 6- or 12-layer stacking sequences are also observed in the lower temperature phases of CuBiP_2_Se_6_.^32^ This ABC stacking is responsible for the helical arrangement of the atoms along the stacking direction, and as discussed further below, has an important influence on the vibrational modes of AgCrP_2_Se_6_.

**Figure 1:**
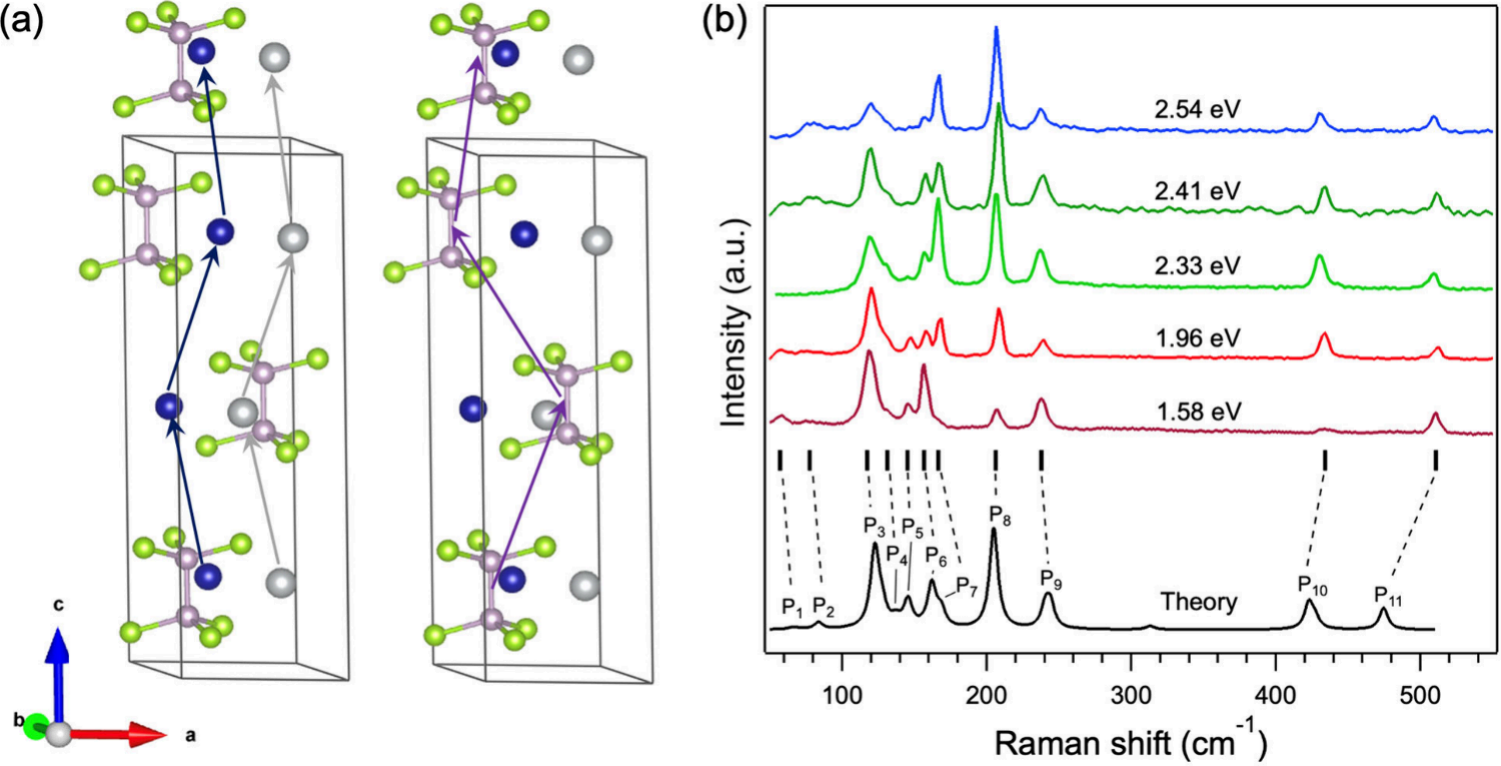
Structure and multiexcitation Raman spectra from AgCrP_2_Se_6_. (a) Schematic showing 4 layers of AgCrP_2_Se_6_ so as to better visualize full translational symmetry along the stacking direction. The arrows indicate a helical arrangement of the Ag^+^ and Cr^3+^ ions and P_2_Se_6_ atom groups along the *c* axis. The Ag, Cr, P, and Se atoms are depicted in gray, blue, purple and green, respectively. (b) Experimentally measured multiexcitation (1.58–2.54 eV) Raman spectra collected at room temperature from bulk AgCrP_2_Se_6_ crystals along with the calculated spectrum (bottom trace). The solid vertical lines denote the experimentally measured peaks (P_1_–P_11_) and are connected to the calculated peaks by the dashed lines.

The trigonal crystal system of AgCrP_2_Se_6_ belongs to the D_3_ point group [*P*3_1_12 (No. 151) space group], which has three irreducible representations of A_1_, A_2_, and E. Its primitive cell contains 30 atoms, resulting in 90 phonons at the Γ point of the Brillouin zone. Of these, 87 phonons are optical and can be decomposed into 15 A_1_, 14 A_2_, and 29 doubly degenerate E symmetry phonons (Γ_vib_ = 15 A_1_ + 14 A_2_ + 2 × 29 E). The A_1_ and E phonons are Raman-active. At room temperature, owing to mode degeneracies and other factors such as sample quality and point or line defects, we were not able to measure all 44 Raman-active modes. Our experimental measurements observed 11 Raman modes (5 A_1_ and 6 E), whose frequencies are listed in Table 1. Unpolarized room temperature Raman spectra from bulk AgCrP_2_Se_6_, collected with multiple laser excitation energies (*E*_laser_ = 1.58, 1.96, 2.33, 2.4, and 2.54 eV), are shown in Figure 1b, together with the spectrum calculated using density functional theory (DFT; bottom spectrum in Figure 1b). In all, 11 Raman modes can be observed in the Raman spectra and are labeled P_1_–P_11_. The A_1_ or E symmetries of the modes were confirmed by collecting linearly polarized Raman spectra. Figure S2 shows angle-dependent colinearly and cross-linearly polarized spectra; the corresponding polar plots for several peaks (P_3_–P_11_) are shown in Figures S3 and S4. The 11 experimentally measured peaks are compared to theory in Figure 1b by the solid vertical lines underneath the spectrum collected with 1.58 eV excitation. All 11 peaks are captured and labeled in the calculated spectrum and connected to the experimentally measured peak labels with dashed lines. Differences between the theoretical and measured Raman peaks, between 0.4 and 11.4 cm^–1^ for P_1_–P_10_ and a relatively larger value for P_11_, are attributed to the use of the Perdew–Burke–Ernzerhof (PBE) exchange-correlation functional. A better agreement between experimental and theoretically calculated frequencies might be achieved by employing a higher level of theory but is beyond the scope of this study.

**Table 1:** Comparison Between Measured and Calculated Raman Peak Frequencies (cm^–1^) in AgCrP_2_Se_6_ Along with Their Corresponding Mode Symmetries^a^

Raman peak	P_1_	P_2_	P_3_	P_4_	P_5_	P_6_	P_7_	P_8_	P_9_	P_10_	P_11_
experiment	**65**	83.5	120	129	**147.5**	158	166.3	208.1	**239.3**	434.4	511.1
theory	**65.4**	83.6	122.3	137.7	**145.6**	162	168.5	204.8	**243.9**	423	474.5
mode symmetry	**E**	E	A_1_	A_1_	**E**	A_1_	E	A_1_	**E**	E	A_1_

a The three helical modes P_1_, P_5_, and P_9_ are indicated in bold font.

The phonon eigenvectors for the 11 Raman modes are shown in Figure 2. Of the 11 modes, 3 (P_1_, P_5_, and P_9_) are helical, exhibiting E symmetry, and following the helical arrangement of the atoms, as shown in Figure 1a. Videos showing animations of the helical atomic displacements are included as Supplementary Video S1, Video S2, and Video S3. P_1_ corresponds to helical vibrations of the Ag^+^ ions, and P_5_ and P_9_ correspond to helical vibrations of the Cr^3+^ ions. P_5_ also involves in-plane vibrations of the Se atoms. For the remaining nonhelical modes, P_3_ corresponds to in-plane shear-like vibrations of the Se atoms, P_8_ is a Se in-plane breathing mode, and P_6_ is an out-of-plane breathing mode of the Se atoms along the *c* axis and perpendicular to the layer stacking direction. The peaks denoted as P_2_, P_4_, and P_7_ correspond to Se atom vibrations with both in-plane and out-of-plane components. The highest frequency modes P_10_ and P_11_ correspond to in-plane and out-of-plane vibrations of the P atoms, respectively. The 11 Raman-active modes measured experimentally along with the DFT-calculated mode frequencies and their corresponding symmetries are listed in Table 1.

**Figure 2:**
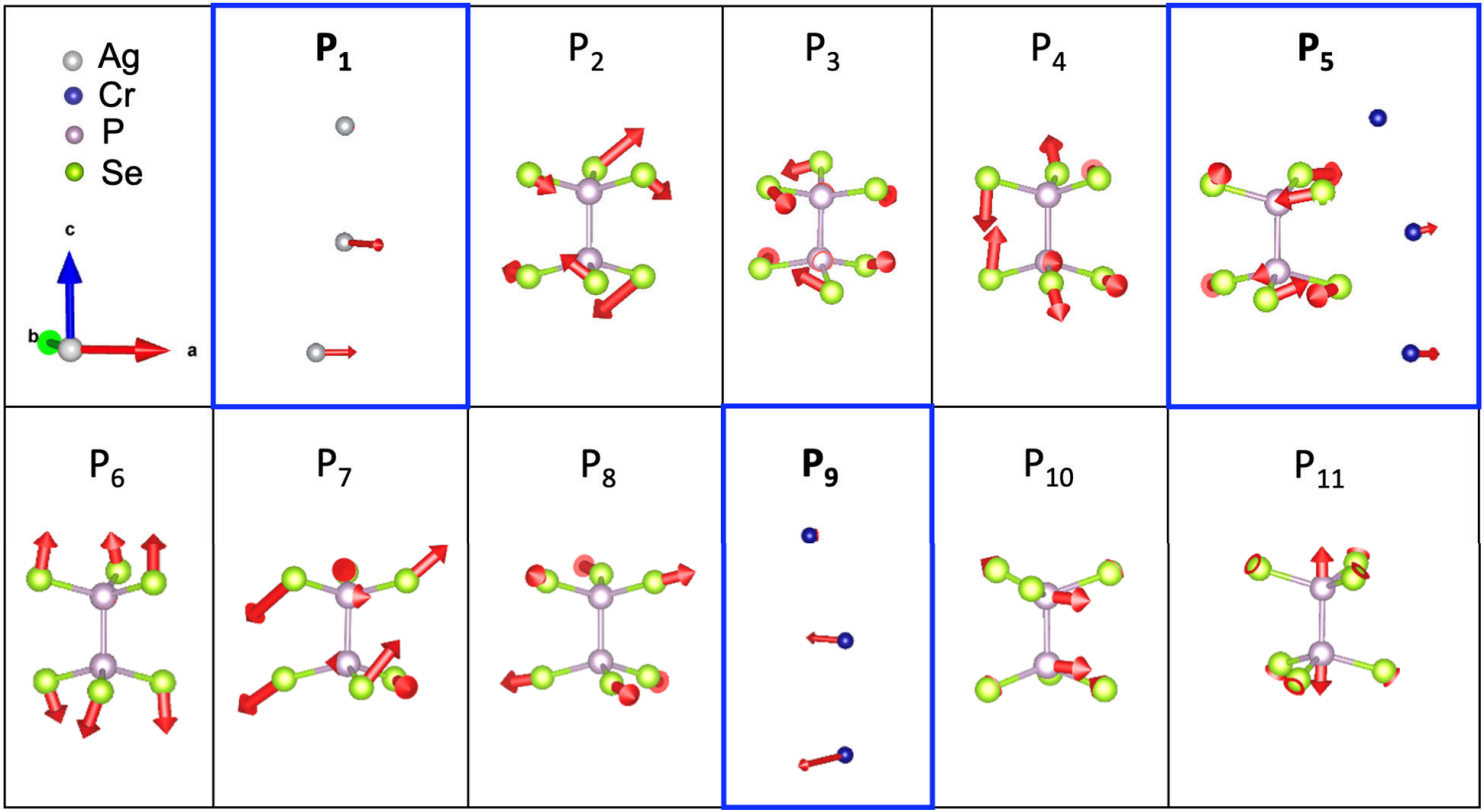
Atomic displacements for the Raman modes. Phonon eigenvectors for the 11 observed Raman peaks (P_1_–P_11_) in AgCrP_2_Se_6_. The Ag, Cr, P, and Se atoms are in silver, blue, violet, and green, respectively. The three helical modes (P_1_, P_5_, and P_9_) are highlighted by the blue boxes.

The presence of helical vibrations in AgCrP_2_Se_6_ is confirmed by measuring circularly polarized Raman spectra. Figure 3a shows circularly polarized Raman spectra collected from a bulk AgCrP_2_Se_6_ crystal using *E*_laser_ = 1.58 eV (785 nm). Left circularly polarized (LCP, σ^+^) incident light was achieved by manually inserting a half waveplate in combination with a quarter waveplate prior to the objective lens in our micro-Raman setup (the optical layout of the circular polarization measurement setup is shown in Figure S5), while the half waveplate was moved out of the beam path to achieve right circularly polarized (RCP, σ^–^) excitation. In our experimental setup, the backscattered light is directed through the notch filter and is polarized parallel or perpendicular, thus giving us co-circularly and cross-circularly polarized configurations (σ^+^σ^+^ or σ^–^σ^+^, respectively). Figure 3a shows a clear difference in intensities of P_1_, P_5_, and P_9_ between RCP and LCP excitations. While the other peaks do not vary in intensity with RCP or LCP excitation, the intensities of P_1_, P_5_, and P_9_ are higher in the RCP spectrum. The variations in intensity due to differences in absorption of RCP or LCP light confirms their helical nature.^33^ The notch filter in our experimental setup also allows us to measure both Stokes and anti-Stokes peaks; we observed similar differences in intensities between the RCP and LCP spectra in the anti-Stokes region (Figure S6). We could not confirm this result with another wavelength on the same flake.

**Figure 3:**
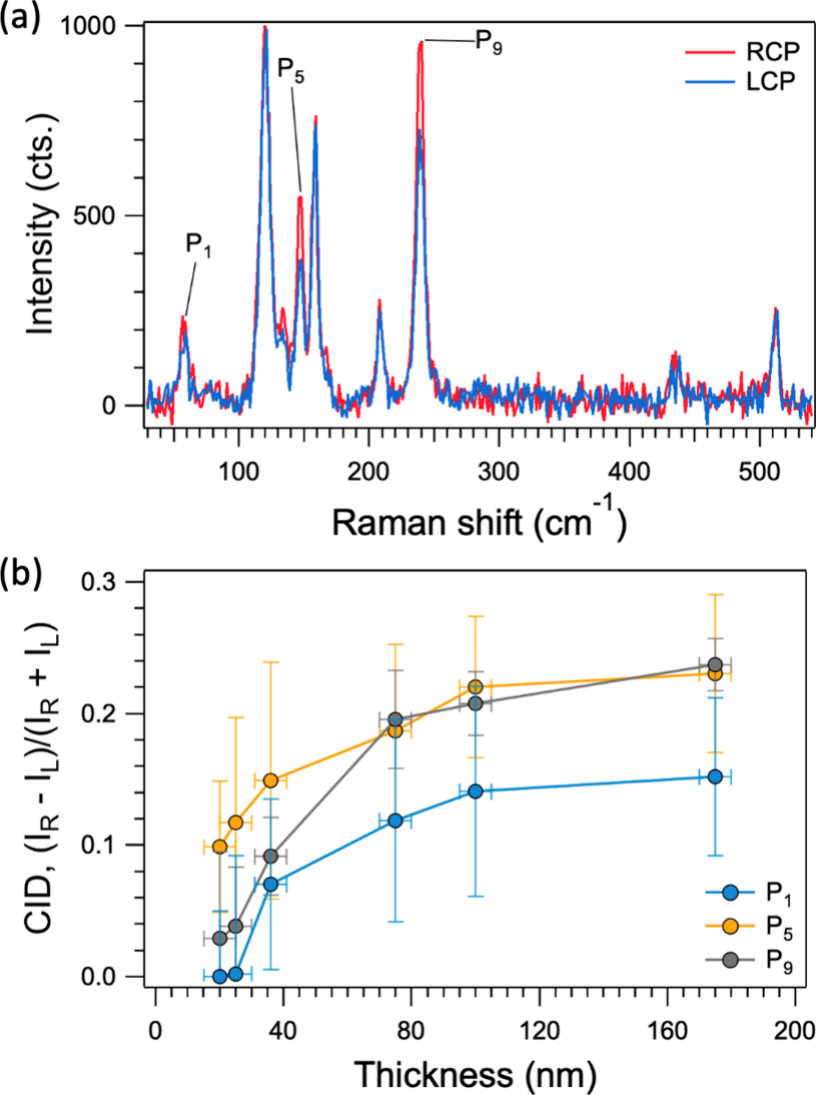
Evidence for chiral phonons in the Raman spectra. (a) Circularly polarized Raman spectra from bulk AgCrP_2_Se_6_, collected with 785 nm (1.58 eV) excitation. (b) Circular intensity difference as a function of the flake thickness for the three chiral modes P_1_, P_5_, and P_9_.

Chiral phonons (i.e., helical or circular vibrating phonon modes associated with angular momenta) have been observed previously in chiral crystals such as α-quartz,^34^ tellurium,^35^ and HgS.^36^ The chiral phonons in these materials are degenerate E modes with opposite phonon pseudoangular momenta (PAM, *l*_ph_ = ±1) unlike the A phonon modes, which have one-dimensional irreducible representation with *l*_ph_ = 0.^37^ The condition of *l*_ph_ = ±1 results in a splitting of the degenerate E modes (typically by a few cm^–1^) at low wavevectors away from the Brillouin zone center, enabling their observation with circularly polarized Raman scattering, which probes phonons near the zone center. In AgCrP_2_Se_6_, the Raman phonons satisfy *C*_3_ rotational symmetry, namely, $C_{3}u\left(r\right)=e^{\left(-i\frac{2\pi }{3}l_{\mathrm{ph}}\right)}u\left(r\right)$ , with *u*(*r*) a phonon eigenvector and *l*_ph_ = 0, ±1 the PAM for the phonon.^24,38^ The PAM of phonons with a general wavevector *q* can be estimated by analyzing their corresponding eigenvectors.^39^ First, a basis set is introduced for right- and left-handed circular modes in the *xy* plane, such that $\left|R_{j}\rangle =\frac{1}{\sqrt{2}}\right|...,1,i,0,...\rangle$ and $\left|L_{j}\rangle =\frac{1}{\sqrt{2}}\right|...1,-i,0,...\rangle$ . The projection of the phonon eigenvector |*u*⟩ to the basis is given by α_*j*_^R^ = ⟨*u*|*R*_*j*_⟩ and α_*j*_^L^ = ⟨*u*|*L*_*j*_⟩. The PAM for the *j*th atom of the phonon is given by |α_*j*_^R^|^2^ – |α_*j*_^L^|^2^ and for the crystal by *s*_ph_ = ∑_*j*=1_^*N*^(|α_*j*_^R^|^2^ – |α_*j*_^L^|^2^), with *N* the number of atoms in the unit cell.^38,39^ The calculated PAM values along the high-symmetry lines in the Brillouin zone are shown in Figure 4. Chiral phonons are found at the Γ point as well as near the high-symmetry points K (^1^/_3_, ^1^/_3_, 0) and H (^1^/_3_, ^1^/_3_, ^1^/_2_). Parts b–d of Figure 4 show the calculated splitting of frequencies of the three helical modes P_1_, P_5_, and P_9_, along with the calculated PAM. The splitting of the modes near the Γ point is very small (∼0.1 cm^–1^) and smaller than the resolution of our measurements. However, P_1_ and P_5_ exhibit PAM along the ΓA direction and P_9_ has PAM along the ΓK direction. The appearance of PAM clearly shows that these modes are chiral and their origin can be attributed to the crystal structure through the *C*_3_ screw symmetry along the stacking direction (Figure 1a). It is also worth noting that we do not see any differences in the peak intensities with LCP and RCP excitation in the spectra from the related materials AgInP_2_Se_6_ and AgInP_2_S_6_ (Figure S7). Both AgInP_2_Se_6_ and AgInP_2_S_6_ exhibit AB layer stacking,^40^ suggesting that the structural anisotropy caused by the ABC layer stacking in AgCrP_2_Se_6_ is uniquely responsible for the presence of the chiral phonon modes.

**Figure 4:**
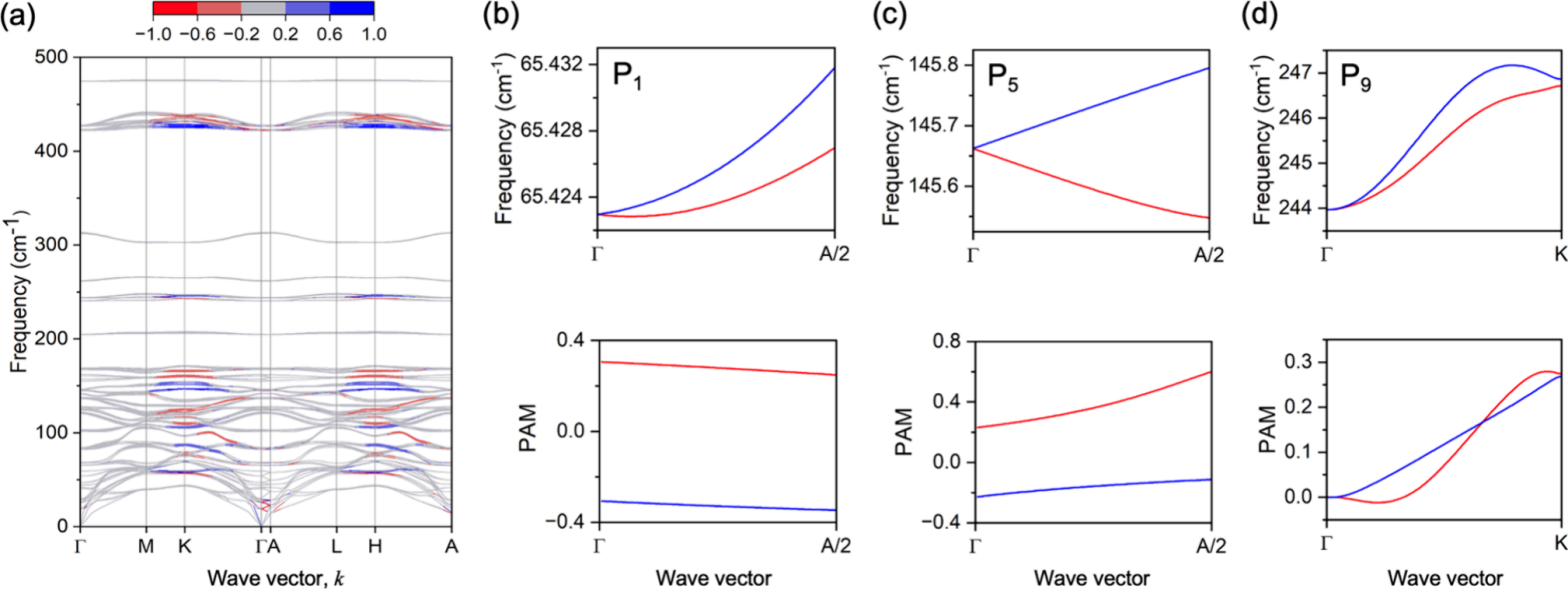
Phonon pseudo angular momentua (PAM). (a) Calculated PAM, coded by color for AgCrP_2_Se_6_ along high-symmetry lines. (b–d) Upper panels showing higher magnification views of the dispersions of P_1_, P_5_, and P_9_, with a small degree of splitting between the degenerate E modes. The lower panels show PAM for the split two branches. Our calculated splitting near Γ between the modes is less than 0.1 cm^–1^, well under the spectral resolution of our instruments, and the PAM calculations show that P_1_, P_5_, and P_9_ are chiral phonon modes.

The differences in spectral intensities with RCP and LCP excitation also prompted us to measure the Raman optical activity (ROA) for the three chiral modes. The ROA is defined as the difference in Raman scattering for RCP and LCP excitations, and its strength is estimated in the form of the circular intensity difference, CID = (*I*_R_ – *I*_L_)/(*I*_R_ + *I*_L_).^33,41^ *I*_R_ and *I*_L_ are the peak intensities corresponding to RCP and LCP excitation, respectively. Note that, in order to estimate *I*_R_ and *I*_L_, the circularly polarized Raman spectra were collected by placing a freshly cleaved AgCrP_2_Se_6_ crystal onto a Si substrate and the two spectra were normalized by considering the response of the Si substrate to the RCP and LCP excitation (under the same measurement conditions). In addition, we accounted for the loss in intensity of the LCP light due to the additional half waveplate in the excitation beam path. The CIDs for the three chiral modes P_1_, P_5_, and P_9_ (obtained from the Stokes Raman peaks for the spectrum plotted in Figure 3a) are 0.15, 0.21, and 0.20, respectively. In contrast, the CID values for the nonchiral modes are an order of magnitude lower (0.012–0.014). The CID values for the three chiral modes are very high compared to what is typically observed for chiral materials, i.e., on the order of 10^–3^.^41^ Recently, circularly polarized Raman studies on 2D ReS_2_ and ReSe_2_ ^22,23^ reported similarly large CID values that were attributed to quantum interference effects. Note that, in ReS_2_ and ReSe_2_, the phonon modes are not helical/chiral but include a combination of in-plane and out-of-plane vibrations of the Re and chalcogen atoms. Thus, our measured CID values for intrinsically chiral phonon modes in AgCrP_2_Se_6_ are ostensibly the highest for a 2D material.

Next, in order to investigate the thickness dependence of the CIDs in AgCrP_2_Se_6_, we mechanically exfoliated flakes down to a minimum thickness of 20 nm onto Si/SiO_2_ (285 nm of oxide) substrates, followed by collection of circularly polarized Raman spectra. The thickness of the exfoliated flakes was confirmed using atomic force microscopy. For each thickness, the RCP and LCP spectra were normalized in the same manner as that described above for the bulk crystal. Figure 3b shows the CIDs for P_1_, P_5_, and P_9_ as a function of thickness. Even with the sizable error bars, all three CIDs can be observed to decrease with thickness. This decrease can be attributed to a reduction in interlayer coupling with decreasing flake thickness. To verify this, we simulated the thickness dependence of the interlayer distance and interlayer binding energy. DFT calculations indicate that the interlayer distance increased from 6.673 to 6.681 Å, and the interlayer binding energy decreased from 66.13 to 65.54 meV/atom when the thickness is decreased from 30 to 24 layers (from 20.1 to 16.1 nm). The trends also support the notion that the ABC stacking is crucial to the occurrence of the chiral phonon modes, and that the disruption of the ABC stacking with decreasing thickness results in the lower CIDs. Raman spectra from even thinner flakes were difficult to obtain owing to the drastically reduced intensities and increased laser-induced degradation. Nonetheless, collectively the appearance of chiral phonons and their thickness dependences hint at the potential to manipulate them through moiré engineering or ion intercalation. Future experiments using ultrafast pump–probe spectroscopy techniques may enable us to gain further understanding into these chiral phonons and their dynamics.

### Excitation-Energy-Dependent Mode Intensities

Going back to Figure 1b, it is clear that the intensities of several Raman peaks depend strongly on *E*_laser_ and that this dependence varies for each mode. Interestingly, the nonchiral Raman modes exhibit the greatest dependence on the excitation energy, with some peaks disappearing completely for certain values of *E*_laser_. This can be seen more clearly in Figure 5, which plots the normalized intensities of the four nonchiral modes that display the greatest variations in intensity (the excitation-energy-dependent intensities of P_3_–P_11_ peaks are shown in Figure S8). Note that the multiexcitation spectra plotted in Figure 1b were collected by placing the crystal on a Si substrate and normalized by accounting for the wavelength-dependent absorption of the Si. Additionally, we were unable to measure the excitation intensity dependence of the lowest frequency modes (P_1_ and P_2_) due to instrumental limitations (lack of low-frequency cutoff filters for all excitations).

**Figure 5:**
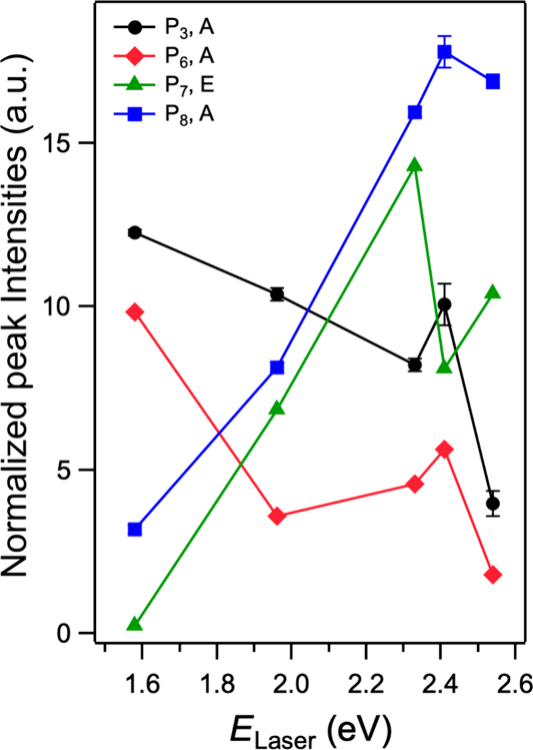
Excitation-energy-dependent Raman intensities. Normalized peak intensities for P_3_, P_6_, P_7_, and P_8_ as a function of excitation laser energy.

Three features can be gleaned from Figures 5 and S8. First, the normalized intensities of P_7_ and P_8_ increase considerably with energy and are maximum at *E*_laser_ = 2.41 and 2.33 eV, respectively; the intensity of P_8_ is over a factor of 5 larger at *E*_laser_ = 2.41 eV compared to its intensity at 1.58 eV. Second, the intensities of P_3_ and P_6_ are the highest for our lowest excitation energy (1.58 eV) and decrease with increasing *E*_laser_. The third feature observed in Figures 5 and S8 is that the strong excitation energy dependence occurs for both E and A_1_ symmetry modes. Curiously, all four peaks that exhibit a strong excitation energy dependence (P_3_, P_7_, P_6_, and P_8_) correspond mainly to vibrations of the Se atoms. Our previous calculations^25^ demonstrated that the Se p orbitals have large contributions to the valence and conduction bands. This could account for our observation of a larger dependence of the intensity on the excitation energy for the Se vibrational modes than for the higher frequency modes (P_10_ and P_11_; cf. Figure S8) that mainly involve vibrations of the P atoms.

The dependence of Raman peak intensities on *E*_laser_ typically arises because of resonance with an electronic state, where, instead of a virtual transition of the absorbed photon, there is a real electronic transition. Such increases in intensities of some Raman peaks at certain laser energies corresponding to excitonic transitions have been reported in MoSe_2_ ^42^ and MoTe_2_.^20^ In order to investigate this more closely, we calculated the optical absorbance spectrum for AgCrP_2_Se_6_, which is plotted in Figure 6a. Additionally, we measured the reflectance spectrum from a bulk crystal, as shown in Figure 6b. The dashed vertical lines in Figure 6 indicate all of the laser excitation energies used in this study. The calculated absorbance spectrum in Figure 6a has peaks close to our laser excitation energies. However, these peaks are not sharp or intense. A similar lack of features can be seen in the experimental reflectance spectrum (Figure 6b), with a relatively flat response (27–28%) across the range of our laser energies. The low reflectance also indicates a high absorption of light across the entire visible-wavelength range. We also observed the same dependence of the peak intensities on *E*_laser_ for mechanically exfoliated flakes. The plots in show Raman spectra collected with *E*_laser_ = 1.58, 1.96, and 2.41 eV where the excitation energy dependence of the peak intensities does not depend on the thickness. It is well-known that the electronic structure of 2D materials depends strongly on their thickness and that, in general, a reduction in thickness is accompanied by an increase in the band gap of the material.^43^ The lack of sharp resonances at our laser energies as well as the independence of the peak intensities on flake thickness together suggest that resonance Raman enhancement is unlikely to be the primary reason for the our excitation-energy-dependent observations (Figure 5).

**Figure 6:**
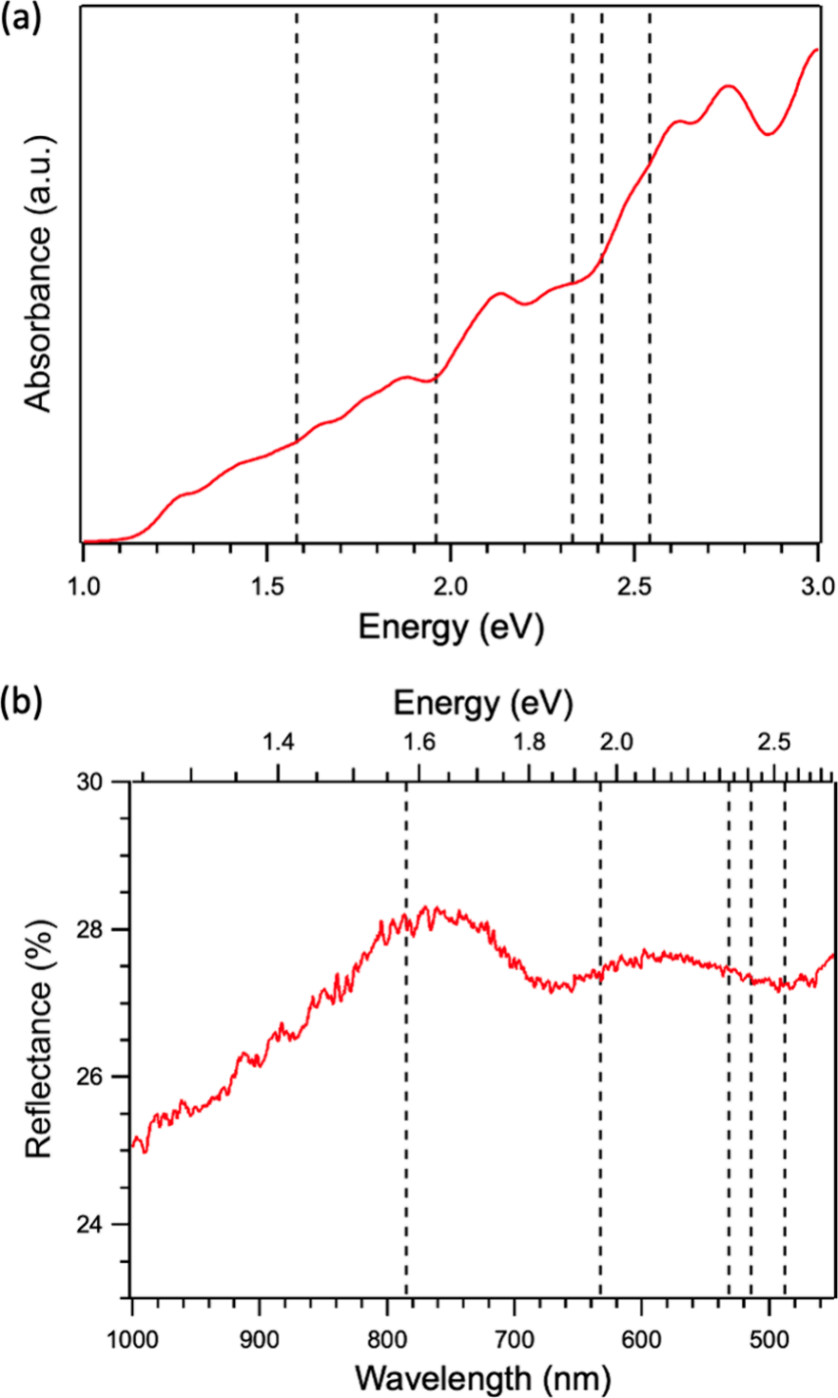
Optical absorption of AgCrP_2_Se_6_. (a) Calculated absorption and (b) measured reflectance curves from AgCrP_2_Se_6_. The vertical dashed lines indicate all of the laser excitation energies used in this study.

An alternate reason for mode-selective excitation-energy-dependent Raman intensities could be the strength of the electron-phonon coupling. Such enhancements have been reported before in other 2D materials like ReS_2_ ^19^ and PdSe_2_,^44^ where the structural anisotropy results in strong excitation energy dependences for peak intensities and cannot be accounted for by resonance with the excitation laser. We previously showed that, among the 2D metal selenophosphates that have been synthesized thus far, AgCrP_2_Se_6_ possesses the lowest volume per P_2_Se_6_ unit (222 Å^3^; cf. Figure 1 in ref 24). The lower volume could lead to a larger overlap between the electron densities of the Se and the metal cations, thus enhancing charge transfer and electron–phonon coupling. We also observed such structure-induced variations in electron/spin–phonon coupling in MPS_3_ materials (M = Ni, Co, Fe, and Mn), where the lowest unit cell volume of NiPS_3_ leads to a strong interaction between the S atoms and Ni^2+^ ions, and thus a higher degree of electron–phonon coupling.^45^ Indeed, a closer look at the calculated projected density of states (PDOS; Figure S10) of AgCrP_2_Se_6_ reveals that the Ag 4d bands hybridize with the Se 4p bands close to the valence band maximum, while the Cr 3d bands hybridize with the Se 4p bands close to the conduction band minimum. The PDOS analyses also indicate a value of 3.97e for Cr^3+^, which assumes a formal 3d occupation number of 3 as per the electronic configuration of Cr. Furthermore, Bader charge analyses indicate a charge transfer of 1.72e from the [P_2_Se_6_]^4–^ units to the Cr^3+^ ions, consistent with self-doping due to charge transfer between the Se and Cr^3+^ ions and similar to what has been reported previously for NiPS_3_ ^46^ (see Figure S10 and the accompanying discussion). Our analysis thus hints at the strong influence of electron–phonon coupling on the Raman peak intensities. Interestingly, temperature-dependent measurements show that the Raman peak frequencies and widths follow the typical anharmonic decay processes^47,48^ of optical phonons (see Figure S11 and the accompanying discussion) and are strongly affected by phonon–phonon interactions rather than electron–phonon interactions.^49–51^ Our analysis of the anharmonic coefficients indicates that a higher degree of anharmonicity in some of the Raman modes, warranting further studies with respect to the thermal properties of AgCrP_2_Se_6_. Moreover, multiexcitation spectral measurements using tunable laser sources as well as calculations of excitation-energy-dependent spectra that include electron–phonon coupling effects may shed more light on our observations.

### Unusual Anti-Stokes Peak Intensities

Last, we report unusual trends in the anti-Stokes intensities in AgCrP_2_Se_6_. In the anti-Stokes scattering process, incident light absorption into the material results in the absorption/annihilation of phonons rather than the creation of phonons like in the Stokes process. As a result, the anti-Stokes Raman scattered light gains energy compared to the laser excitation energy. The phonon absorption occurs from excited state phonon populations, and thus the anti-Stokes process is closely correlated with temperature, with the anti-Stokes peak intensity increasing with temperature. The ratio of intensities between the anti-Stokes and Stokes Raman peaks (*I*_AS_/*I*_S_) is a commonly used metric that is related to the temperature (*T*) according to the following relationship $\frac{I_{\mathrm{AS}}}{I_{S}}={\left(\frac{\omega_{L}+\omega_{i}}{\omega_{L}-\omega_{i}}\right)}^{4}\;\exp\left(-\frac{\hbar \omega_{i}}{kT}\right)$ ,^52^ where ω_L_ and ω_*i*_ are the laser frequency and the frequency of the *i* ^th^ mode, respectively, *ℏ* is the reduced Planck’s constant, and *k* is the Boltzmann constant.

Figure 7a shows the Stokes and anti-Stokes regions (positive and negative frequencies, respectively) of unpolarized Raman spectra from bulk AgCrP_2_Se_6_ crystals, collected with *E*_laser_ = 1.58, 1.96, and 2.41 eV. The spectra in Figure 7a were collected at room temperature with the lowest laser powers that produced measurable signals and reasonable signal/noise ratios (64, 105, and 68 μW for *E*_laser_ = 1.58, 1.96, and 2.41 eV, respectively). We fit the Raman peaks in the spectra in Figure 7a with Voigt lineshapes to obtain the anti-Stokes and Stokes intensities (*I*_AS_ and *I*_S_, respectively), whose ratios are plotted in Figure 7b against *E*_laser_ for four Raman peaks: P_3_, P_6_, P_8_, and P_9_. The figure shows that the measured *I*_AS_/*I*_S_ ratios are highest for *E*_laser_ = 1.58 eV and decrease with increasing *E*_laser_ for all four peaks.

**Figure 7:**
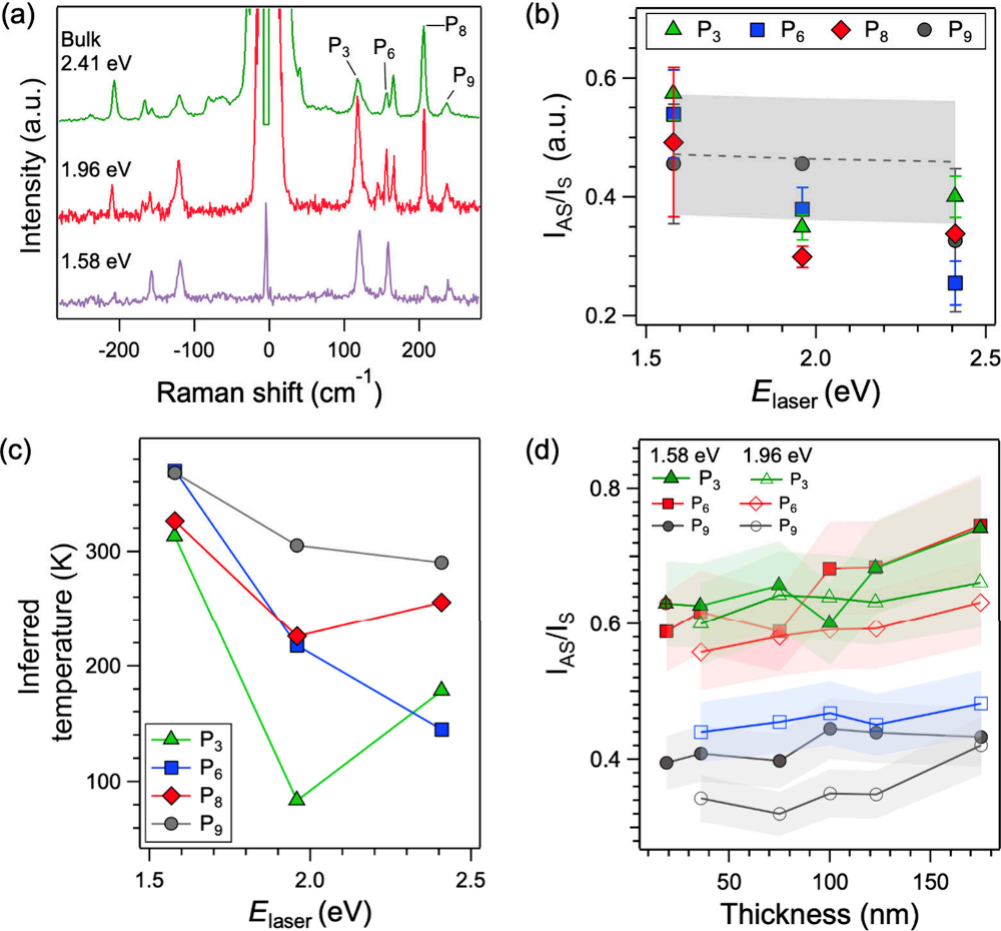
Anti-Stokes Raman spectra. (a) Room temperature multiexcitation Raman spectra in the Stokes and anti-Stokes regions from AgCrP_2_Se_6_ crystals. (b) *I*_AS_/*I*_S_ ratios for four Raman modes (P_3_, P_6_, P_8_, and P_9_) as a function of *E*_laser_. The dashed line is the calculated *I*_AS_/*I*_S_ ratios corresponding to room temperature (298 K), and the gray shaded area represents the spread in values for the three laser excitation energies. (c) Temperatures calculated from the measured *I*_AS_/*I*_S_ ratios, plotted against *E*_laser_. (d) *I*_AS_/*I*_S_ ratios as a function of the AgCrP_2_Se_6_ flake thickness. The filled and open data points correspond to *E*_laser_ = 1.58 and 1.96 eV, respectively. The errors in the estimation of the *I*_AS_/*I*_S_ ratios are plotted as error surfaces.

Excitation-energy-dependent variations in *I*_AS_/*I*_S_ ratios can arise from resonance effects, where the excitation laser is in resonance with either the Stokes or anti-Stokes photons. In other words, the resonance occurs for energies that are greater or lower than the laser energy by the phonon energy for anti-Stokes and Stokes photons, respectively. In our case the highest energy phonon mode in Figure 7a (P_9_ at ∼240 cm^–1^) corresponds to 29.7 meV higher or lower than the laser energy. As shown in Figure 6a,b, we do not see any sharp features in the optical spectrum of AgCrP_2_Se_6_ between 500 and 800 nm that could account for resonances with the Stokes or anti-Stokes scattered photon energies. Moreover, AgCrP_2_Se_6_ is an indirect gap semiconductor (band gap ∼ 1.24 eV),^25^ and we do not expect resonance at the band gap energy. Thus, even though our lowest excitation energy (1.58 eV) is considerably higher than the band gap, the trend of increasing *I*_AS_/*I*_S_ ratios with decreasing laser energy cannot be attributed to laser excitation with energies close­(r) to resonance. The unusual laser energy dependence of the *I*_AS_/*I*_S_ ratios are likely attributable to a different origin.

Also plotted in Figure 7b are the theoretically expected (according to the above relationship) *I*_AS_/*I*_S_ ratios at 298 K. The dashed line in Figure 7b is the average calculated *I*_AS_/*I*_S_ ratio, and the shaded region represents the spread in values for the various laser energies. While there are slight variations in the laser energy dependence of the *I*_AS_/*I*_S_ ratios between the peaks, on the whole the *I*_AS_/*I*_S_ ratios are slightly above the expected room temperature values for *E*_laser_ = 1.58 eV and lower than expected for *E*_laser_ = 1.96 and 2.41 eV. The lower *I*_AS_/*I*_S_ ratios signify an apparent cooling of the phonon modes. Again, using the above relationship, we calculated the temperatures from the *I*_AS_/*I*_S_ ratios. Apart from the highest frequency P_9_ mode at ∼240 cm^–1^, the temperatures inferred from the *I*_AS_/*I*_S_ ratios for the other peaks (P_3_, P_6_, and P_8_) range from 83 to 256 K, all significantly below room temperature. The inferred temperatures obtained from the *I*_AS_/*I*_S_ ratios for the four Raman modes are plotted in Figure 7c. We also measured the *I*_AS_/*I*_S_ ratios in mechanically exfoliated flakes as a function of thickness, and they are plotted in Figure 7d. On the whole, the *I*_AS_/*I*_S_ ratios decrease with the thickness for both of the measured laser excitations (1.58 and 1.96 eV).

The phenomenon of laser-induced cooling by the removal of phonons is well-known and has been mainly observed in ultrafast Raman spectroscopy measurements.^53,54^ However, our measurements were performed under steady state conditions, wherein the energy gained by removing phonons must be somehow dissipated. Laser-induced cooling in steady state (under continuous-wave excitation) was previously reported for individual semiconducting single-walled carbon nanotubes where the low *I*_AS_/*I*_S_ ratios were attributed to a phonon-assisted interband relaxation and luminescence emission process.^55^ However, we do not observe any luminescence at energies other than across the band edge in AgCrP_2_Se_6_,^25^ suggesting that this mechanism is not responsible for our observed low *I*_AS_/*I*_S_ ratios for *E*_laser_ = 1.96 and 2.41 eV. To gain more insights, we performed laser-power- and temperature-dependent Raman measurements. The *I*_AS_/*I*_S_ ratios exhibit an exponential dependence on laser powers (Figure S12), following the Boltzmann phonon population distribution equation and implying that the anti-Stokes phonon populations are thermally driven with increasing laser powers. Interestingly, for the highest laser power (1.78 mW) with the 1.58 eV excitation, the calculated temperature from the *I*_AS_/*I*_S_ ratios is over 1100 K (Figure S13), which is above the typical decomposition temperature of this class of materials (1000–1100 K).^30,56^ We confirmed the structural stability of the material by collecting a Raman spectrum after lowering the power. We also measured temperature-dependent Raman spectra and saw similar trends across our whole temperature range (85–300 K); i.e., the *I*_AS_/*I*_S_ ratios were consistently higher in the measured data compared to the calculated values according to the Boltzmann equation for *E*_laser_ = 1.58 eV but slightly lower or equal to the calculated values for *E*_laser_ = 1.96 eV ().

To address the origin of the unusual Stokes and anti-Stokes intensities in AgCrP_2_Se_6_ and its excitation energy dependence, we first note that the calculated absorption spectrum and the experimental reflectance spectrum show increasing light absorption at shorter wavelengths. Second, we turn back to the discussion in the previous section, namely, the hybridization between the Se and Cr orbitals resulting in charge transfer between the Se and Cr^3+^ ions. We propose that the greater light absorption at higher laser excitation energies heightens this charge transfer, and the depopulation/annihilation of the vibrational modes (and hence lower *I*_AS_/*I*_S_ ratios) compensates for the energy needed for this charge transfer. Such a phenomenon was recently observed in steady state surface-enhanced Raman spectroscopy measurements from aromatic thiols adsorbed on plasmonic gold nanoparticles,^57^ wherein a Marcus charge transfer process was postulated as the reason for the apparent cooling of the molecule by ∼60 K. Finally, the decreasing *I*_AS_/*I*_S_ ratios with thickness (Figure 7d) mirror that of the CIDs (Figure 3b) and could be attributed to a reduction in the interlayer ABC coupling, causing a reduction in this charge transfer. Further ultrafast spectroscopic measurements as well as excitation-energy-dependent computations may help to explain our observations.

## Conclusions

We have computed and measured the Raman spectra from bulk and exfoliated AgCrP_2_Se_6_, and observe several unusual features: (1) Three of the 11 Raman-active modes are chiral and were confirmed with circularly polarized Raman measurements and DFT calculations of the PAM. These modes exhibit large circular intensity differences that decrease with thickness in the exfoliated flakes. (2) Several modes exhibit distinct excitation-energy-dependent intensities. (3) We observe low anti-Stokes Raman peak intensities with increasing laser excitation energy, which results in an apparent cooling of the material by up to 220 K. We attribute these anomalies to the ABC layer stacking structure of AgCrP_2_Se_6_ and to the smaller octahedral cage surrounding the metal cations that causes hybridization between the Se and Ag/Cr electron densities, resulting in charge transfer and strongly affecting the electron–phonon coupling. Coupled with our previous observation of sharp defect-induced luminescence at ∼696 nm,^25^ this work establishes AgCrP_2_Se_6_ as an exciting new 2D material for a variety of optical and phononics applications.

## Methods and Experimental Procedures

AgCrP_2_Se_6_ single crystals were synthesized by combining Ag foil (Johnson Matthey, 99.99%), H_2_-reduced Cr powder (Aldrich, >99.5%), Se shot (Alfa Aesar Puratronic, 99.999%), and P lumps (Alfa Aesar Puratronic, 99.999%) together with ∼100 mg of I_2_ (Alfa Aesar ACS, 99.8%) in a sealed quartz ampoule. The ampoule was inserted into a tube furnace which was heated to a temperature of 650 °C and held for 200 h to drive the reaction to completion. The crystals were micaceous and presented as well-faceted distorted hexagons mostly 20–200 μm in diameter, although a few grew to 2 mm and were reserved for magnetic and optical characterization; thicknesses were ∼20–40 μm. We confirmed the stoichiometry and crystal structure of the product by electron-dispersive spectroscopy (Thermo Scientific Ultra Dry EDS spectrometer joined with a JEOL JSM-6060 scanning electron microscope), which revealed the composition to be Ag_1.13(8)_Cr_0.94(10)_P_1.87(9)_Se_6.07(10)_, within error to the ideal composition of AgCrP_2_Se_6_.

DFT calculations were performed with the Vienna *ab initio* simulation package (*VASP5.4*), applying the projector-augmented-wave (PAW) potential.^58,59^ The Kohn–Sham equations were solved using a plane wave basis set with an energy cutoff of 500 eV. We employed our experimentally determined structure as the initial structure and optimized it using the Perdew–Burke–Ernzerhof (PBE) exchange-correlation functional,^60^ including the D3 correction^61^ for London dispersion An antiferromagnetic structure was considered, where the spins are in the *ab* plane and align in an antiferromagnetic configuration along the *c* axis, as found in our experiment. We used a 1 × 1 × 2 supercell for the antiferromagnetic structure. The *k*-point sampling was taken as 8 × 8 × 1. Geometries were fully relaxed regarding lattice parameters and interatomic distances until forces were less than 0.001 eV/Å. Using the optimized structure, we utilized the SCAN (strongly constrained and appropriately normed) exchange-correlation functional.^62,63^ The Hubbard correction used for the Cr d orbitals was *U* = 3 eV and *J* = 0. The Raman spectrum of AgCrP_2_Se_6_ was calculated using Phonopy and Phonopy spectroscopy at the PBE+D3 level.^64,65^ The Phonopy package was employed to calculate the zone-center phonon frequencies and phonon eigenvectors of the DFT-optimized structures using the finite-displacement approach. The force constant matrix was constructed in a 2 × 2 × 1 supercell. We simulated the Raman spectrum by averaging the Raman tensor calculated for each Raman-active phonon eigenmode at the zone center.

We performed mechanical exfoliation of the AgCrP_2_Se_6_ single crystals using a PDMS stamp. A crystal was first thinned with several Scotch tape exfoliation cycles, followed by thinning onto the PDMS stamp. We then exfoliated the flakes onto cleaned Si/SiO_2_ (285 nm of SiO_2_) substrates by placing the PDMS stamp onto the substrate and applying pressure (using ∼90 N force) for 20 min followed by slowly peeling the stamp off of the substrate. Several solvent rinses were then carried out to remove as much residue as possible.

Room temperature Raman spectra were collected in two Renishaw inVia Raman microscopes. One of the inVia Raman microscopes was outfitted with a low-frequency module (Coherent/Ondax THz Raman probe) that uses fiber optics to couple 785 nm laser excitation and to direct the scattered light into the inVia spectrometer. The optical layout for this setup is shown in Figure S3. The other inVia Raman microscope was used for 633, 514.5, and 488 nm excitation. The spectra were collected by focusing the laser on the exfoliated and bulk crystals through a 50× or 100× objective lenses. Additionally, all spectra were collected with low excitation powers (<0.1 mW) to minimize laser-induced surface degradation. The CIDs were calculated by baseline subtraction, followed by Lorentzian line shape fitting. The multiexcitation spectra were collected on thick exfoliated AgCrP_2_Se_6_ flakes on Si/SiO_2_ substrates, and the spectra were normalized by the Si peak intensities. Reflectance spectra (between 200 and 1100 nm) were collected with a Craic microspectrophotometer. We performed these measurements by placing a freshly cleaved bulk crystal on silicon substrates and by focusing the excitation source (Xe lamp) through a 74× objective lens. The reference used to obtain the reflectance of the bulk sample is a Ag mirror (Thorlabs), with >95% reflectance between 450 and 1100 nm. Additional Raman spectra from both the anti-Stokes and Stokes regions were collected with a commercial Horiba LabRAM HR Evolution confocal microscope using a set of ultralow-frequency filters with excitation lasers of 2.33 eV (532 nm) and 1.96 eV (633 nm) usinga backscattering geometry [1800 lines/mm grating, 100 μm confocal hole, 50× LWD objective (0.8 N.A.), and 1 μm laser spot size]. All measurements were performed at room temperature under ambient conditions.

## Supplementary Material









## Notes

Additional supporting figures and discussion including linearly and circularly polarized Raman spectra, a schematic of our circular polarization, optical layout, excitation-energy-dependent intensities, thickness-dependent spectra for three laser excitations, PDOS, temperature-dependent peak frequencies and linewidths, anti-Stokes/Stokes intensity ratios for multiple excitations, and inferred temperatures as a function of the laser power (PDF)
Video showing an animation of the helical atomic displacement (MP4)
Video showing an animation of the helical atomic displacement (MP4)
Video showing an animation of the helical atomic displacement (MP4)

## References

[1] Rudenko A. N., Katsnelson M. I. (2024). Anisotropic Effects in Two-Dimensional Materials. 2D Mater..

[2] Li X., Liu H., Ke C., Tang W., Liu M., Huang F., Wu Y., Wu Z., Kang J. (2021). Review of
Anisotropic 2D Materials:
Controlled Growth, Optical Anisotropy Modulation, and Photonic Applications. Laser Photonics Rev..

[3] Vannucci L., Petralanda U., Rasmussen A., Olsen T., Thygesen K. S. (2020). Anisotropic
Properties of Monolayer 2D Materials: An Overview from the C2DB Database. J. Appl. Phys..

[4] Li Z., Xu B., Liang D., Pan A. (2020). Polarization-Dependent Optical Properties
and Optoelectronic Devices of 2D Materials. Research.

[5] Wang C., Zhang G., Huang S., Xie Y., Yan H. (2020). The Optical
Properties and Plasmonics of Anisotropic 2D Materials. Advanced Optical Materials.

[6] Zhao X., Li Z., Wu S., Lu M., Xie X., Zhan D., Yan J. (2024). Raman Spectroscopy
Application in Anisotropic 2D Materials. Adv
Electron. Mater..

[7] Nemilentsau A., Low T., Hanson G. (2016). Anisotropic 2D Materials for Tunable Hyperbolic Plasmonics. Physical review letters.

[8] Ruta F. L., Zhang S., Shao Y., Moore S. L., Acharya S., Sun Z., Qiu S., Geurs J., Kim B. S. Y., Fu M., Chica D. G., Pashov D., Xu X., Xiao D., Delor M., Zhu X.-Y., Millis A. J., Roy X., Hone J. C., Dean C. R., Katsnelson M. I., van Schilfgaarde M., Basov D. N. (2023). Hyperbolic Exciton Polaritons in
a van Der Waals Magnet. Nat. Commun..

[9] Wang H., Qian X. (2017). Giant Optical Second
Harmonic Generation in Two-Dimensional Multiferroics. Nano Lett..

[10] Puebla S., D’Agosta R., Sanchez-Santolino G., Frisenda R., Munuera C., Castellanos-Gomez A. (2021). In-Plane Anisotropic
Optical and Mechanical Properties
of Two-Dimensional MoO_3_. npj 2D Mater.
Appl..

[11] Li L., Han W., Pi L., Niu P., Han J., Wang C., Su B., Li H., Xiong J., Bando Y., Zhai T. (2019). Emerging In-plane
Anisotropic Two-dimensional Materials. InfoMat.

[12] Ling X., Huang S., Hasdeo E. H., Liang L., Parkin W. M., Tatsumi Y., Nugraha A. R. T., Puretzky A. A., Das P. M., Sumpter B. G., Geohegan D. B., Kong J., Saito R., Drndic M., Meunier V., Dresselhaus M. S. (2016). Anisotropic
Electron–Photon and Electron–Phonon Interactions in
Black Phosphorus. Nano Lett..

[13] Ribeiro H. B., Pimenta M. A., De Matos C. J., Moreira R. L., Rodin A. S., Zapata J. D., De Souza E. A., Castro Neto A. H. (2015). Unusual
Angular Dependence of the Raman Response in Black Phosphorus. ACS Nano.

[14] Resende G. C., Ribeiro G. A. S., Silveira O. J., Lemos J. S., Brant J. C., Rhodes D., Balicas L., Terrones M., Mazzoni M. S. C., Fantini C., Carvalho B. R., Pimenta M. A. (2021). Origin of the Complex
Raman Tensor Elements in Single-Layer Triclinic ReSe_2_. 2D Mater..

[15] Wolverson D., Crampin S., Kazemi A. S., Ilie A., Bending S. J. (2014). Raman Spectra
of Monolayer, Few-Layer, and Bulk ReSe_2_: An Anisotropic
Layered Semiconductor. ACS Nano.

[16] Gong Y., Zhao Y., Zhou Z., Li D., Mao H., Bao Q., Zhang Y., Wang G. P. (2022). Polarized
Raman Scattering of In-Plane
Anisotropic Phonon Modes in α-MoO_3_. Advanced Optical Materials.

[17] Šiškins M., Lee M., Alijani F., van Blankenstein M. R., Davidovikj D., van der Zant H. S. J., Steeneken P. G. (2019). Highly Anisotropic Mechanical and
Optical Properties of 2D Layered As_2_S_3_ Membranes. ACS Nano.

[18] Song Q., Pan X., Wang H., Zhang K., Tan Q., Li P., Wan Y., Wang Y., Xu X., Lin M., Wan X., Song F., Dai L. (2016). The In-Plane Anisotropy of WTe_2_ Investigated by Angle-Dependent and Polarized Raman Spectroscopy. Sci. Rep..

[19] McCreary A., Simpson J. R., Wang Y., Rhodes D., Fujisawa K., Balicas L., Dubey M., Crespi V. H., Terrones M., Hight Walker A. R. (2017). Intricate Resonant Raman Response in Anisotropic ReS_2_. Nano Lett..

[20] Song Q., Wang H., Pan X., Xu X., Wang Y., Li Y., Song F., Wan X., Ye Y., Dai L. (2017). Anomalous
In-Plane Anisotropic Raman Response of Monoclinic Semimetal 1 T́-MoTe
2. Sci. Rep.

[21] Goldstein T., Chen S.-Y., Tong J., Xiao D., Ramasubramaniam A., Yan J. (2016). Raman Scattering and Anomalous Stokes-Anti-Stokes
Ratio in MoTe_2_ Atomic Layers. Sci.
Rep.

[22] Zhang S., Mao N., Zhang N., Wu J., Tong L., Zhang J. (2017). Anomalous
Polarized Raman Scattering and Large Circular Intensity Differential
in Layered Triclinic ReS_2_. ACS Nano.

[23] Zhang S., Huang J., Yu Y., Wang S., Yang T., Zhang Z., Tong L., Zhang J. (2022). Quantum Interference
Directed Chiral Raman Scattering in Two-Dimensional Enantiomers. Nat. Commun..

[24] Zhu H., Yi J., Li M.-Y., Xiao J., Zhang L., Yang C.-W., Kaindl R. A., Li L.-J., Wang Y., Zhang X. (2018). Observation
of Chiral Phonons. Science.

[25] Susner M. A., Conner B. S., Rowe E., Siebenaller R., Giordano A., McLeod M. V., Ebbing C. R., Bullard T. J., Selhorst R., Haugan T. J., Jiang J., Pachter R., Rao R. (2024). Structural, Magnetic, and Optical
Properties of the van Der Waals
Antiferromagnet AgCrP_2_Se_6_. J. Phys. Chem. C.

[26] Susner M. A., Chyasnavichyus M., McGuire M. A., Ganesh P., Maksymovych P. (2017). Metal Thio-
and Selenophosphates as Multifunctional van Der Waals Layered Materials. Adv. Mater..

[27] Bousquet E., Fava M., Romestan Z., Gómez-Ortiz F., McCabe E. E., Romero A. H. (2025). Structural Chirality and Related
Properties in the Periodic Inorganic Solids: Review and Perspectives. J. Phys.: Condens. Matter.

[28] Wang T., Sun H., Li X., Zhang L. (2024). Chiral Phonons:
Prediction, Verification,
and Application. Nano Lett..

[29] Pfeiff R., Kniep R. (1993). Preparation of Quaternary
Selenodiphosphates­(IV) from Halide Melts:
The Crystal Structure of CuAl­[P_2_Se_6_]. Zeitschrift für Naturforschung B.

[30] Susner M. A., Belianinov A., Borisevich A., He Q., Chyasnavichyus M., Demir H., Sholl D. S., Ganesh P., Abernathy D. L., McGuire M. A., Maksymovych P. (2015). High-Tc Layered Ferrielectric Crystals
by Coherent Spinodal Decomposition. ACS Nano.

[31] Klingen W., Ott R., Hahn H. (1973). Uber Die Darstellung Und Eigenschaften von Hexathio-und
Hexaselenohypodiphosphaten. Zeitschrift für
anorganische und allgemeine Chemie.

[32] Gave M. A., Bilc D., Mahanti S. D., Breshears J. D., Kanatzidis M. G. (2005). On the Lamellar Compounds CuBiP_2_Se_6_, AgBiP2Se6 and AgBiP2S6. Antiferroelectric
Phase Transitions Due
to Cooperative Cu+ and Bi3+ Ion Motion. Inorg.
Chem..

[33] Er E., Chow T. H., Liz-Marzán L.
M., Kotov N. A. (2024). Circular
Polarization-Resolved Raman Optical Activity: A Perspective on Chiral
Spectroscopies of Vibrational States. ACS Nano.

[34] Pine A. S., Dresselhaus G. (1971). Raman Spectra and Lattice Dynamics of Tellurium. Phys. Rev. B.

[35] Ishito K., Mao H., Kobayashi K., Kousaka Y., Togawa Y., Kusunose H., Kishine J., Satoh T. (2023). Chiral Phonons: Circularly Polarized
Raman Spectroscopy and Ab Initio Calculations in a Chiral Crystal
Tellurium. Chirality.

[36] Ishito K., Mao H., Kousaka Y., Togawa Y., Iwasaki S., Zhang T., Murakami S., Kishine J., Satoh T. (2023). Truly Chiral Phonons
in α-HgS. Nat. Phys..

[37] Streib S. (2021). Difference
between Angular Momentum and Pseudoangular Momentum. Phys. Rev. B.

[38] Zhang, L. ; Niu, Q. Chiral Phonons at High-Symmetry Points in Monolayer Hexagonal Lattices. Phys. Rev. Lett. 2015, 115 (11). 10.1103/PhysRevLett.115.115502. 26406841

[39] Basak S., Ptok A. (2022). Ab Initio Study of
Chiral Phonons in Ternary YAlSi Compound. Crystals.

[40] Wang X., Du K., Liu W., Hu P., Lu X., Xu W., Kloc C., Xiong Q. (2016). Second-Harmonic
Generation in Quaternary
Atomically Thin Layered AgInP_2_S_6_ Crystals. Appl. Phys. Lett..

[41] Barron L., Buckingham A. (1971). Rayleigh and Raman Scattering from Optically Active
Molecules. Mol. Phys..

[42] Nam D., Lee J.-U., Cheong H. (2015). Excitation
Energy Dependent Raman
Spectrum of MoSe_2_. Sci. Rep.

[43] Roldán R., Silva-Guillén J. A., López-Sancho M. P., Guinea F., Cappelluti E., Ordejón P. (2014). Electronic
Properties of Single-layer and Multilayer Transition Metal Dichalcogenides
MX_2_ (M= Mo, W and X= S, Se). Annalen
der Physik.

[44] Luo W., Oyedele A. D., Mao N., Puretzky A., Xiao K., Liang L., Ling X. (2022). Excitation-Dependent Anisotropic
Raman Response of Atomically Thin Pentagonal PdSe_2_. ACS Phys. Chem. Au.

[45] Rao R., Selhorst R., Siebenaller R., Giordano A. N., Conner B. S., Rowe E., Susner M. A. (2024). Mode-Selective
Spin-Phonon Coupling
in van Der Waals Antiferromagnets. Advanced
Physics Research.

[46] Kim S. Y., Kim T. Y., Sandilands L. J., Sinn S., Lee M.-C., Son J., Lee S., Choi K.-Y., Kim W., Park B.-G., Jeon C., Kim H.-D., Park C.-H., Park J.-G., Moon S. J., Noh T. W. (2018). Charge-Spin Correlation in van Der
Waals Antiferromagnet NiPS_3_. Phys.
Rev. Lett..

[47] Liu F., Parajuli P., Rao R., Wei P. C., Karunarathne A., Bhattacharya S., Podila R., He J., Maruyama B., Priyadarshan G., Gladden J. R., Chen Y. Y., Rao A. M. (2018). Phonon
Anharmonicity in Single-Crystalline SnSe. Phys.
Rev. B.

[48] Rao R., Susner M. A. (2023). Phonon
Anharmonicity in Cu-Based Layered Thiophosphates. Materials Today Communications.

[49] Yang H.-Y., Yao X., Plisson V., Mozaffari S., Scheifers J. P., Savvidou A. F., Choi E. S., McCandless G. T., Padlewski M. F., Putzke C., Moll P. J. W., Chan J. Y., Balicas L., Burch K. S., Tafti F. (2021). Evidence of a Coupled
Electron-Phonon Liquid in NbGe_2_. Nat. Commun..

[50] Plisson, V. M. ; Yao, X. ; Wang, Y. ; Varnavides, G. ; Suslov, A. ; Graf, D. ; Choi, E. S. ; Yang, H.-Y. ; Wang, Y. ; Romanelli, M. ; McNamara, G. ; Singh, B. ; McCandless, G. T. ; Chan, J. Y. ; Narang, P. ; Tafti, F. ; Burch, K. S. Engineering Anomalously Large Electron Transport in Topological Semimetals. Adv. Mater. 2024, 36 (24). 10.1002/adma.202310944. 38470991

[51] Muhammad Z., Hussain G., Islam R., Zawadzka N., Hossain M. S., Iqbal O., Babiński A., Molas M. R., Xue F., Zhang Y., Hasan M. Z., Zhao W. (2024). Electronic Transport
and Interaction of Lattice Dynamics in Topological Nodalline Semimetal
HfAs_2_ Single Crystals. Adv. Funct.
Mater..

[52] Kip B. J., Meier R. J. (1990). Determination
of the Local Temperature at a Sample
during Raman Experiments Using Stokes and Anti-Stokes Raman Bands. Appl. Spectrosc..

[53] Kim J. E., Mathies R. A. (2002). Anti-Stokes Raman
Study of Vibrational Cooling Dynamics
in the Primary Photochemistry of Rhodopsin. J. Phys. Chem. A.

[54] Liu W., Tang L., Oscar B. G., Wang Y., Chen C., Fang C. (2017). Tracking Ultrafast
Vibrational Cooling during Excited-State Proton
Transfer Reaction with Anti-Stokes and Stokes Femtosecond Stimulated
Raman Spectroscopy. J. Phys. Chem. Lett..

[55] Baltog I., Baibarac M., Lefrant S. (2008). Optical Cooling of Single-Walled
Carbon Nanotubes as Revealed by Their Anti-Stokes Raman Spectra. J. Phys.: Condens. Matter.

[56] Susner M. A., Chyasnavichyus M., Puretzky A. A., He Q., Conner B. S., Ren Y., Cullen D. A., Ganesh P., Shin D., Demir H., McMurray J. W., Borisevich A. Y., Maksymovych P., McGuire M. A. (2017). Cation-Eutectic Transition via Sublattice Melting in
CuInP_2_S_6_/In_4/3_P_2_S_6_ van Der Waals Layered Crystals. ACS
Nano.

[57] Yu Z., Frontiera R. R. (2023). Ostensible Steady-State Molecular Cooling with Plasmonic
Gold Nanoparticles. ACS Nano.

[58] Kresse G., Furthmuller J. (1996). Efficiency
of ab-initio total energy calculations for
metals and semiconductors using a plane-wave basis set. Computational Materials Science.

[59] Kresse G., Joubert D. (1999). From ultrasoft pseudopotentials
to the projector augmented-wave
method. Physical Review B.

[60] Perdew J. P., Burke K., Ernzerhof M. (1997). Generalized
Gradient Approximation
Made Simple. Phys. Rev. Lett..

[61] Grimme S., Antony J., Ehrlich S., Krieg H. (2010). A consistent and accurate
ab initio parametrization of density functional dispersion correction
(DFT-D) for the 94 elements H-Pu. The Journal
of Chemical Physics.

[62] Sun J., Ruzsinszky A., Perdew J. P. (2015). Strongly Constrained and Appropriately
Normed Semilocal Density Functional. Phys. Rev.
Lett..

[63] Sun J., Remsing R. C., Zhang Y., Sun Z., Ruzsinszky A., Peng H., Yang Z., Paul A., Waghmare U., Wu X., Klein M. L., Perdew J. P. (2016). Accurate first-principles structures
and energies of diversely bonded systems from an efficient density
functional. Nat. Chem..

[64] Togo A., Tanaka I. (2015). First principles phonon
calculations in materials science. Scripta Materialia.

[65] Skelton J. M., Burton L. A., Jackson A. J., Oba F., Parker S. C., Walsh A. (2017). Lattice dynamics of the tin sulphides
SnS 2, SnS and Sn 2 S 3: vibrational
spectra and thermal transport. Physical Chemistry
Chemical Physics.

