# Supplementary material for: Chiral Phonons and Anomalous Excitation-Energy-Dependent Raman Intensities in Layered AgCrP2Se6
Source: ACS Nano. 2025 Jul 14;19(29):26377–87. doi: 10.1021/acsnano.5c00381 (PMC12981025; doi:10.1021/acsnano.5c00381)
Supplement: Supplementary file 1 [file nn5c00381_si_001.pdf]

Supplementary Information

Chiral Phonons and Anomalous Excitation Energy-dependent Raman Intensities in Layered $AgCrP_2Se_6$

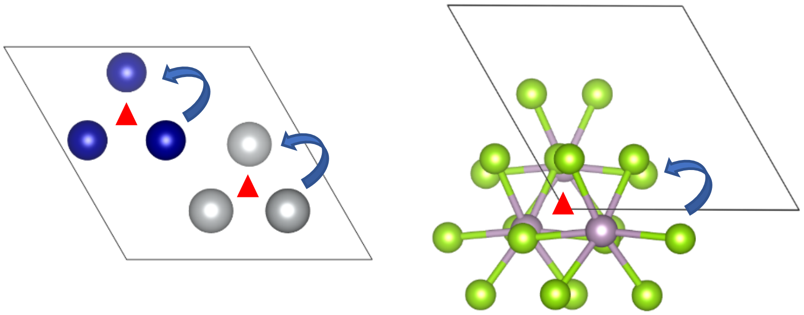

Figure S1. C_3 rotational symmetry on the *ab*-plane with the C_{3_1} screw axes indicated by red triangles. The Ag, Cr, P, and Se atoms are depicted in grey, blue, purple and green, respectively.

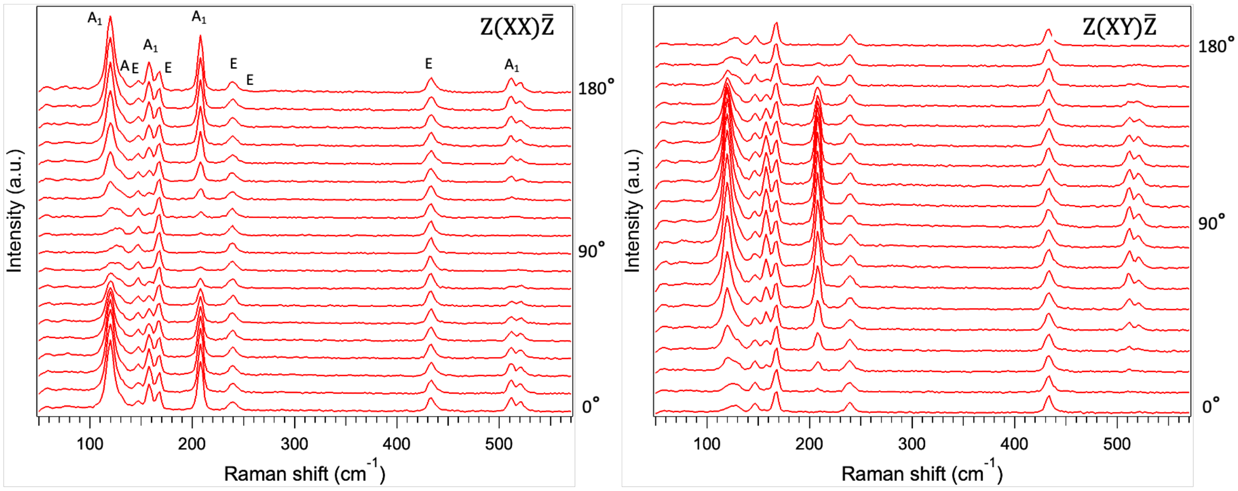

Figure S2. Linearly polarized Raman spectra collected in the backscattering configuration from bulk $AgCrP_2Se_6$ (excitation wavelength 633 nm, 1.96 eV). The out-of-plane A_1 and in-plane E modes can be discerned from the angular dependence of their intensities in the co- and cross-polarized configurations [$Z(XX)\bar{Z}$ and $Z(XY)\bar{Z}$, respectively].

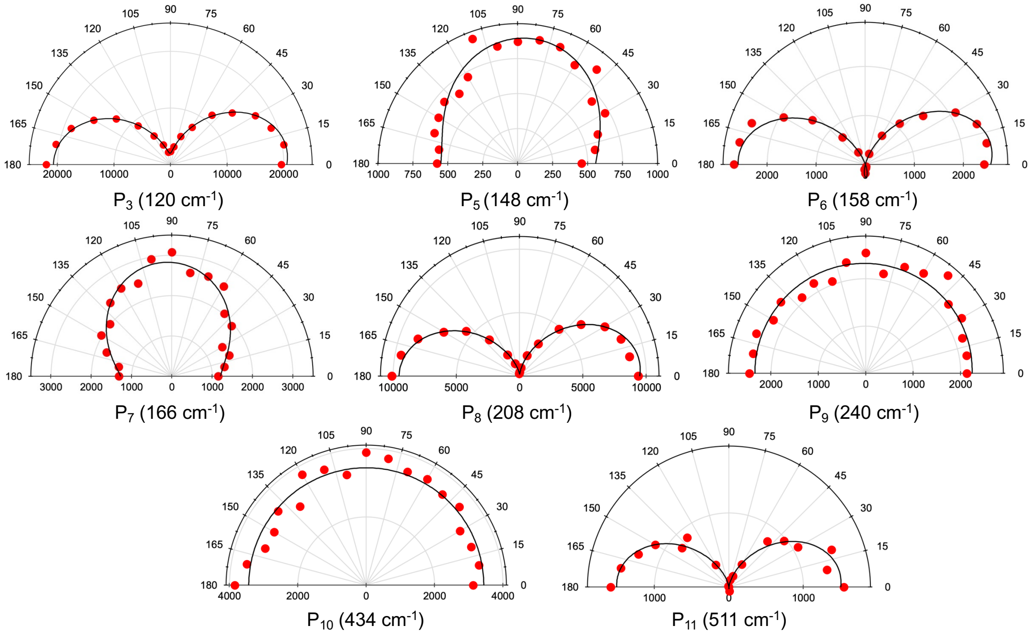

Figure S3. Polar plots for $P_3 - P_{11}$ for the $Z(XX)\bar{Z}$ configuration.

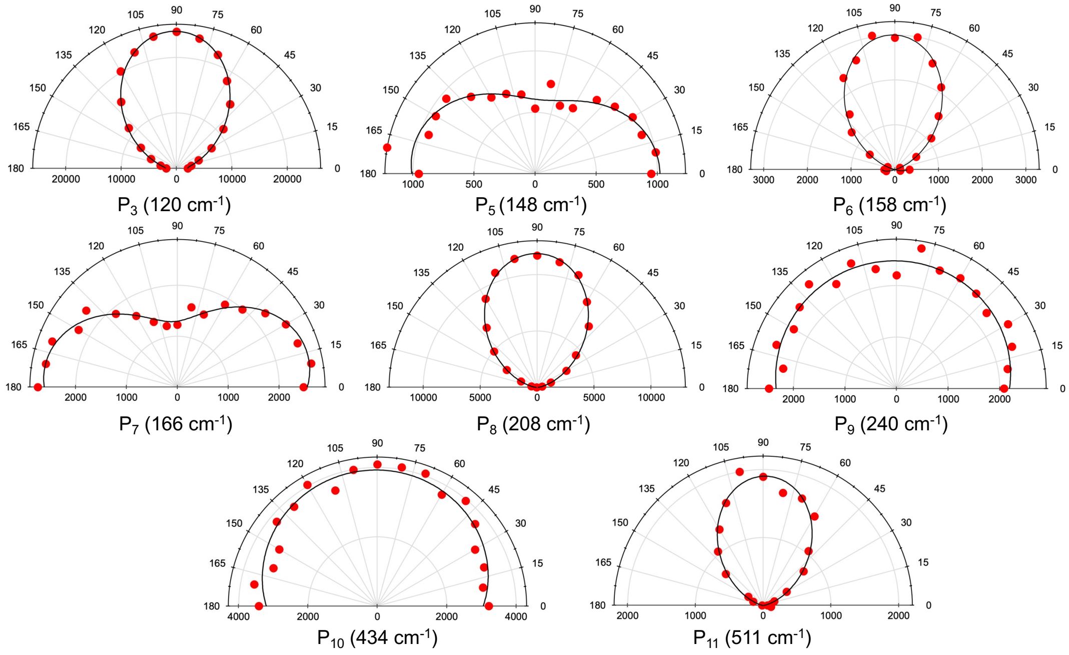

Figure S4. Polar plots for $P_3 - P_{11}$ for the $Z(XY)\bar{Z}$ configuration.

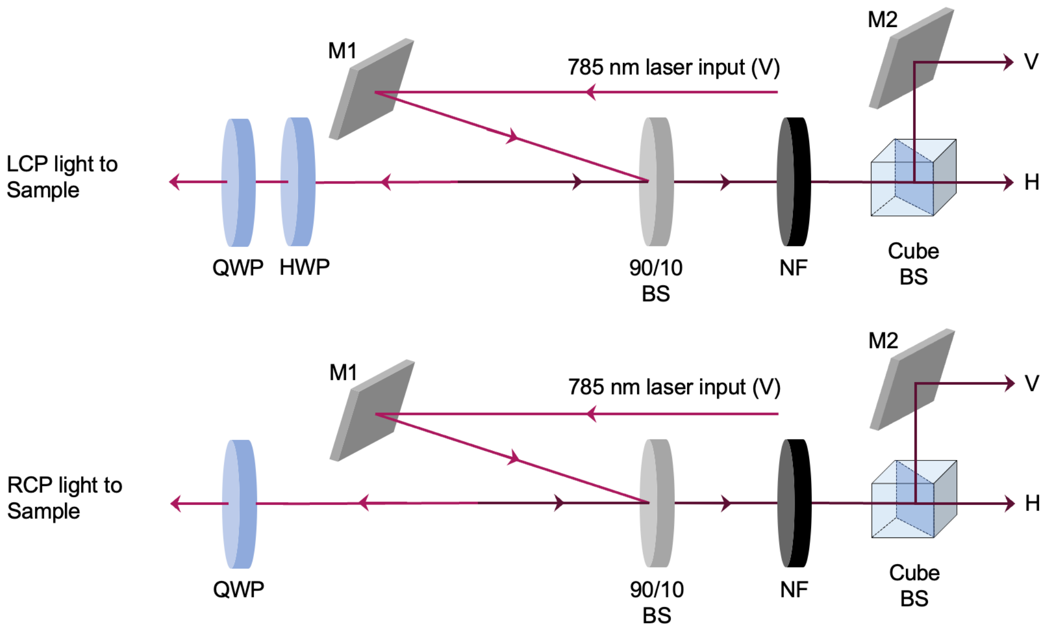

Figure S5. Optical layout showing the configurations for left (top) and right (bottom) circularly polarized excitation. QWP and HWP are the quarter and half waveplates respectively, M1 and M2 are mirrors, NF is the notch filter and the two beamsplitters are labeled BS. In our configuration, the laser excitation is polarized vertically (laboratory coordinates) and directed onto the sample by mirror M1 and through a 90/10 BS. The vertical polarization can be converted to LCP or RCP by manually placing HWP and/or QWP into the beam path. The backscattered light from the sample passes through the same waveplates, which reverses the circular polarization of the scattered light (e.g. incident LCP is converted to scattered RCP). This light passes through the 90/10 BS and NF and is converted into linearly polarized (vertical, V or horizontal, H) light with a polarizing cube BS at the exit port. The scattered light is then taken into the spectrometer using a polarization maintaining optical fiber.

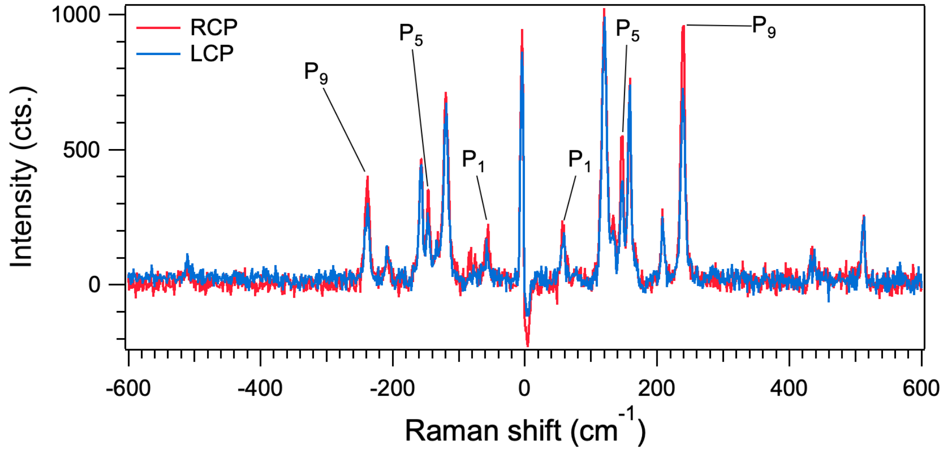

Figure S6. Circularly polarized Raman spectrum from bulk $AgCrP_2Se_6$, showing both anti-Stokes and Stokes regions. We observe similar differences in intensities between LCP and RCP excited spectra for the three helical modes P_1, P_5 and P_9.

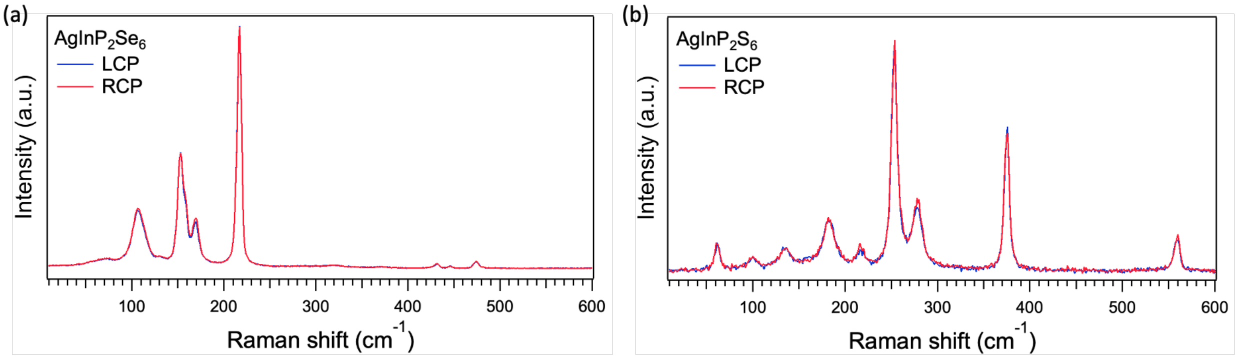

Figure S7. Circularly polarized Raman spectra collected with 1.58 eV (785 nm) excitation from a) $AgInP_2Se_6$ and b) $AgInP_2S_6$ crystals. The near-perfect overlap between the two sets of spectra shows that there is no significant difference in peak intensities with LCP/RCP excitation.

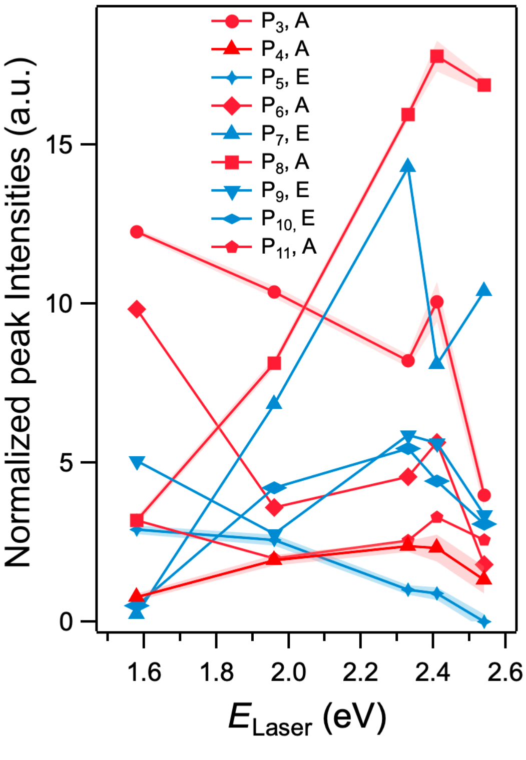

Figure S8 – Normalized peak intensities for P_3 – P_{11} as a function of excitation laser energy. The A and E symmetry modes are plotted in red and blue colored data points, respectively. The errors are shown by surfaces when they are larger than the size of data points.

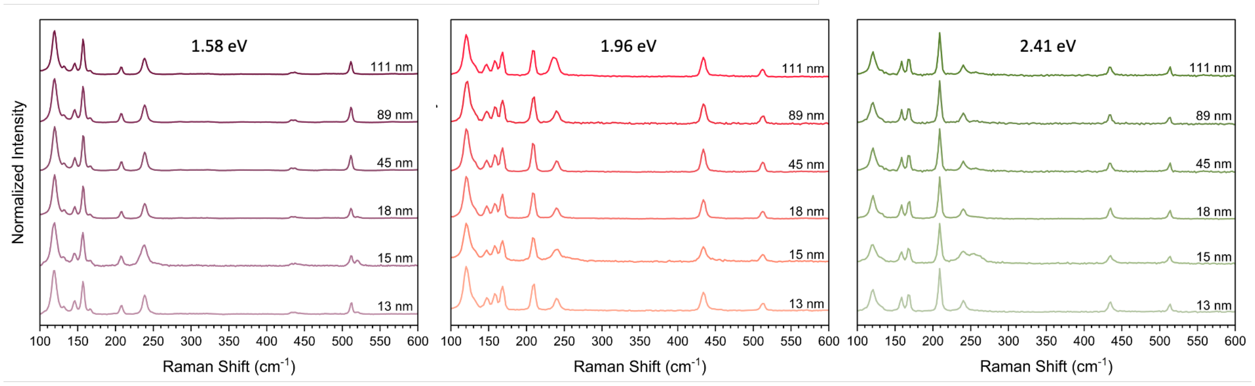

Figure S9. Thickness-dependent Raman spectra from mechanically exfoliated $AgCrP_2Se_6$ flakes, collected with 1.58 (left plot), 1.96 (middle plot) and 2.41 eV (right plot) excitations. Regardless of thickness, the flakes exhibit similar excitation energy-dependent peak intensities.

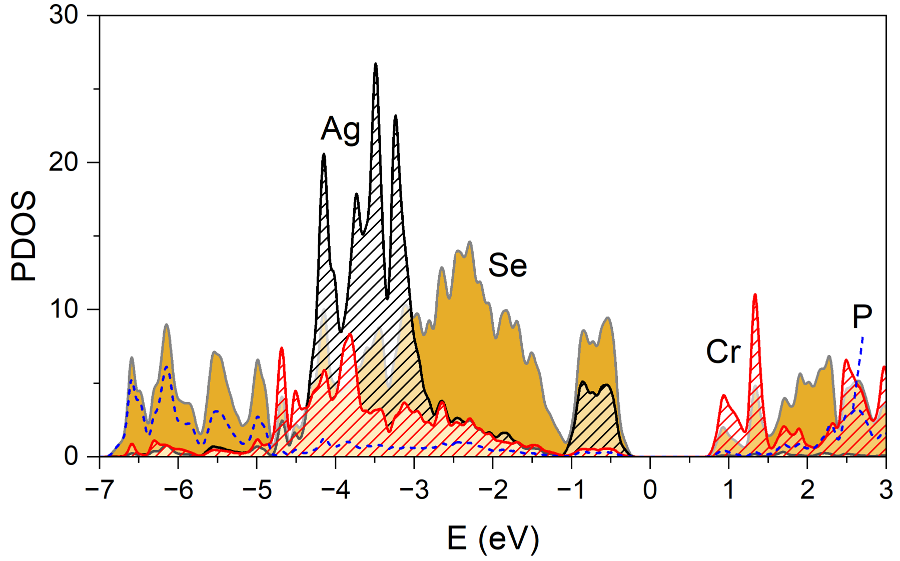

Figure S10. PDOS for the $AgCrP_2Se_6$ antiferromagnetic structure. The DFT calculations used SCAN+U with spin polarization and the D3 van der Waals correction. The red, brown, black, and blue lines denote Cr, Se, and Ag PDOS, respectively. The Fermi level is set at zero.

The projected density of states (PDOS) is shown in Figure S10. The spin up and spin down bands are degenerate, as expected for an antiferromagnetic configuration. The PDOS indicate that the P 3*p* states are mostly further from the Fermi level and hybridize with the Se 4*p* states. The Ag 4*d* bands hybridize with Se 4*p* bands close to the valence band maximum, while close to the conduction band minimum the Cr 3*d* bands hybridize with Se 4*p*. In the energy range of up to 5 eV below the Fermi level, the occupied Ag 4*d* and Cr 3*d* orbitals are located mostly at lower energies than the occupied Se orbitals. A self-doping mechanism for d-levels was derived by crystal field theory, with charge

transfer from a p-shell to a d-level,[1] and was demonstrated for Ni in $NiPS_3$.[2] Here, analysis of the PDOS indicates a value of 3.97e for Cr^{3+}, which assumes a formal 3d occupation number of 3 as per the electronic configuration, thus qualitatively consistent with a self-doping mechanism. However, the value of 9.30e for Ag^{+} does not demonstrate this behavior (formal occupation number 10), noting that evaluation of charge based on the PDOS may not be fully accurate, as dependent on the definition of the Wigner–Seitz radius in the VASP software.[3] We also performed Bader charge analyses to demonstrate charge transfer between $[P_2Se_6]^{4-}$ units and Cr^{3+}. Our analysis indicates values of 10.65e for Ag^{+}, 4.72e for Cr^{3+}, and 47.63e for $[P_2Se_6]^{4-}$, which assumes formal occupation numbers of 10e for Ag^{+}, 3e for Cr^{3+}, and 50 for $[P_2Se_6]^{4-}$ per electronic configuration. Therefore, 0.65e and 1.72e are transferred from $[P_2Se_6]^{4-}$ to Ag^{+} and Cr^{3+}, respectively.

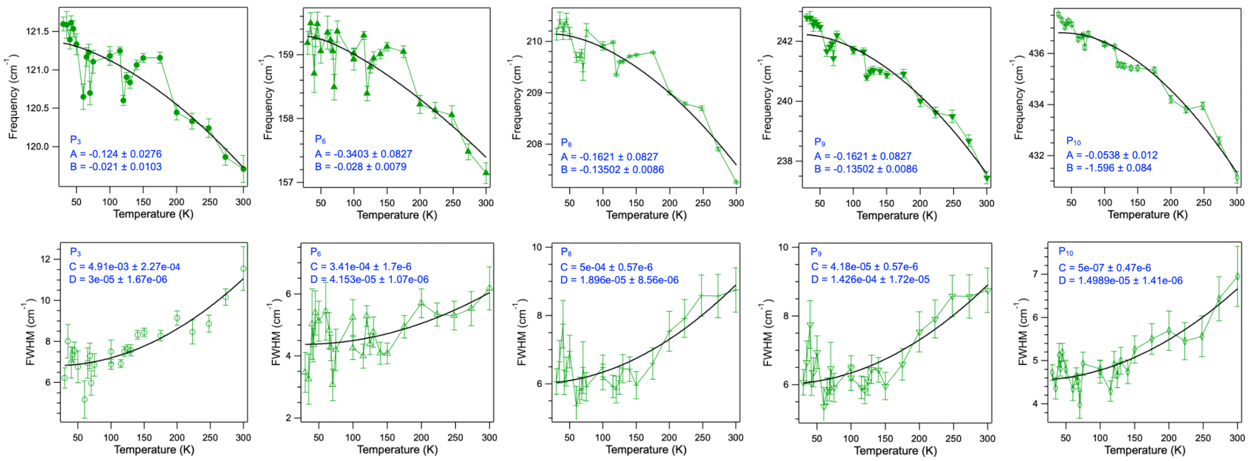

Figure S11. Temperature dependence of frequencies (top row) and widths (bottom row) for peaks P_3, P_6, P_8, P_9 and P_{10}.

We fit the anharmonic temperature-dependent frequencies and widths for several peaks (P_3, P_6, P_8, P_9 and P_{10}) using the following equations –

$$\Delta\omega_j(T) = A\left[1 + \frac{2}{e^x - 1}\right] + B\left[1 + \frac{3}{e^y - 1} + \frac{3}{(e^y - 1)^2}\right]$$

$$\Delta\Gamma_j(T) = \Gamma_0 + C\left[1 + \frac{2}{e^x - 1}\right] + D\left[1 + \frac{3}{e^y - 1} + \frac{3}{(e^y - 1)^2}\right]$$

Here ω_j and Γ_j are the frequency and width of the j'th mode, $x = \hbar\omega_j/2k_BT$, $y = \hbar\omega_j/3k_BT$, $\hbar$ is the reduced Planck constant, k_B is the Boltzmann constant. A, C and B, D are anharmonic coefficients for the decay of the phonon by three- and four-phonon decay processes, respectively. As can be seen in Figure S11, despite the noise in the data (and the discontinuities in frequencies and widths due to the structural phase transitions described in our previous paper,[4] we can fit the data quite well to the anharmonic decay equations described above.

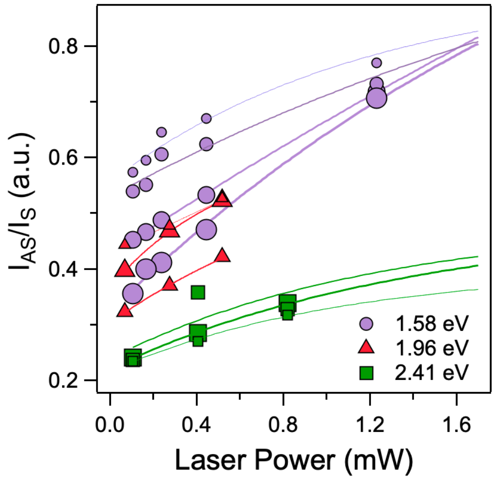

Figure S12. I_{AS}/I_S ratios as a function of laser power for three laser excitation energies. The marker size is proportional to peak frequency and corresponds (from smallest to largest) to P_3, P_6, P_8 and P_9 for E_{laser}=1.58 eV, and for P_3, P_6, and P_8 for E_{laser} = 1.96 and 2.41 eV. The exponential dependences of the I_{AS}/I_S ratios on power show that the phonon populations are thermally driven.

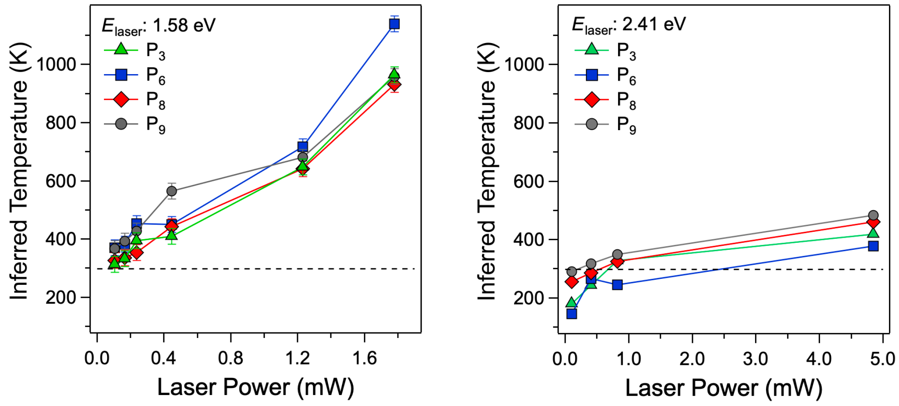

Figure S13. Inferred temperatures vs. laser power for excitation energies of 1.58 (left) and 2.41 eV (right).

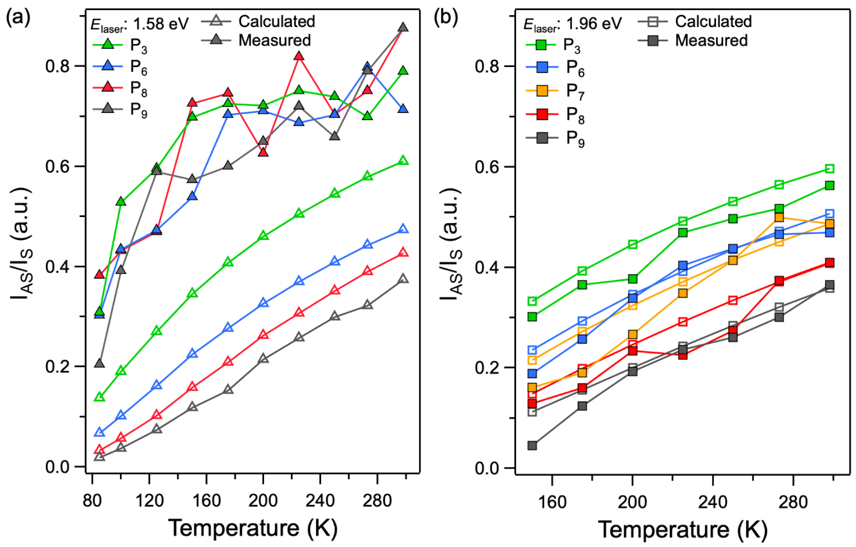

Figure S14. Measured (using E_{laser} = 1.58 eV) and calculated I_{AS}/I_S ratios (filled and empty data points, respectively) as a function of temperature using (a) E_{laser} = 1.58 eV, and (b) E_{laser} = 1.96 eV excitation. The measured values are consistently higher than the expected (calculated) values across the whole temperature range for E_{laser} = 1.58 eV, but they are closer to the calculated values for E_{laser} = 1.96 eV, consistent with the data reported in Figure 7.

References

[1] A. Ushakov, S. Streltsov, D. Khomskii, *Journal of Physics: Condensed Matter* **2011**, *23*, 445601.

[2] S. Y. Kim, T. Y. Kim, L. J. Sandilands, S. Sinn, M.-C. Lee, J. Son, S. Lee, K.-Y. Choi, W. Kim, B.-G. Park, C. Jeon, H.-D. Kim, C.-H. Park, J.-G. Park, S. J. Moon, T. W. Noh, *Phys. Rev. Lett.* **2018**, *120*, 136402.

[3] J. Hafner, G. Kresse, in *Properties of Complex Inorganic Solids*, Springer, **1997**, pp. 69–82.

[4] M. A. Susner, B. S. Conner, E. Rowe, R. Siebenaller, A. Giordano, M. V. McLeod, C. R. Ebbing, T. J. Bullard, R. Selhorst, T. J. Haugan, J. Jiang, R. Pachter, R. Rao, *J. Phys. Chem. C* **2024**, *128*, 4265.
